# The atypical chemokine receptor 2 reduces T cell expansion and tertiary lymphoid tissue but does not limit autoimmune organ injury in lupus-prone B6lpr mice

**DOI:** 10.3389/fimmu.2024.1377913

**Published:** 2024-05-10

**Authors:** Wenkai Xia, Nuru Eltrich, Volker Vielhauer

**Affiliations:** ^1^ Division of Nephrology, Department of Medicine IV, LMU University Hospital, Ludwig-Maximilians-Universität München, Munich, Germany; ^2^ Department of Nephrology, Jiangyin People’s Hospital Affiliated to Nantong University, Jiangyin, China

**Keywords:** systemic lupus erythematosus, lupus nephritis, autoimmune lung disease, chemokines, atypical chemokine receptors

## Abstract

**Introduction:**

The atypical chemokine receptor 2 (ACKR2) is a chemokine scavenger receptor, which limits inflammation and organ damage in several experimental disease models including kidney diseases. However, potential roles of ACKR2 in reducing inflammation and tissue injury in autoimmune disorders like systemic lupus erythematosus (SLE) and lupus nephritis are unknown, as well as its effects on systemic autoimmunity.

**Methods:**

To characterize functional roles of ACKR2 in SLE, genetic Ackr2 deficiency was introduced into lupus-prone C57BL/6lpr (Ackr2-/- B6lpr) mice.

**Results:**

Upon inflammatory stimulation *in vitro*, secreted chemokine levels increased in Ackr2 deficient tubulointerstitial tissue but not glomeruli. Moreover, Ackr2 expression was induced in kidneys and lungs of female C57BL/6lpr mice developing SLE. However, female Ackr2-/- B6lpr mice at 28 weeks of age showed similar renal functional parameters as wildtype (WT)-B6lpr mice. Consistently, assessment of activity and chronicity indices for lupus nephritis revealed comparable renal injury. Interestingly, Ackr2-/- B6lpr mice showed significantly increased renal infiltrates of CD3+ T and B cells, but not neutrophils, macrophages or dendritic cells, with T cells predominantly accumulating in the tubulointerstitial compartment of Ackr2-/- B6lpr mice. In addition, histology demonstrated significantly increased peribronchial lung infiltrates of CD3+ T cells in Ackr2-/- B6lpr mice. Despite this, protein levels of pro-inflammatory chemokines and mRNA expression of inflammatory mediators were not different in kidneys and lungs of WT- and Ackr2-/- B6lpr mice. This data suggests compensatory mechanisms for sufficient chemokine clearance in Ackr2-deficient B6lpr mice *in vivo*. Analysis of systemic autoimmune responses revealed comparable levels of circulating lupus-associated autoantibodies and glomerular immunoglobulin deposition in the two genotypes. Interestingly, similar to kidney and lung CD4+ T cell numbers and activation were significantly increased in spleens of Ackr2-deficient B6lpr mice. In lymph nodes of Ackr2-/- B6lpr mice abundance of activated dendritic cells decreased, but CD4+ T cell numbers were comparable to WT. Moreover, increased plasma levels of CCL2 were present in Ackr2-/- B6lpr mice, which may facilitate T cell mobilization into spleens and peripheral organs.

**Discussion:**

In summary, we show that ACKR2 prevents expansion of T cells and formation of tertiary lymphoid tissue, but is not essential to limit autoimmune tissue injury in lupus-prone B6lpr mice.

## Introduction

Systemic lupus erythematosus (SLE) is a paradigmatic autoimmune disease that presents a broad spectrum of clinical manifestations, of which lupus nephritis is the clinically most important complication. Deposited immune complexes and subsequent complement activation result in renal damage that further releases inflammatory factors. These trigger infiltration of leukocytes into glomerular and tubulointerstitial regions of inflamed kidneys to amplify the renal inflammation and injury (1). Thus, leukocytes accumulation into inflamed kidneys is a critical process in the development of lupus nephritis.

It is well known that chemokines and chemokine receptors mediate infiltration of activated leukocytes into inflamed tissue and contribute to tissue inflammation in SLE (2, 3). The atypical chemokine receptors 2 (ACKR2, previously known as D6 or CCBP2) belongs to the subfamily of atypical chemokine receptors (4). These are non-signaling decoy receptors, which do not induce cell activation, but modulate the chemokine system, e.g. by scavenging, internalizing and degrading bound chemokines, thereby reducing local chemokine concentrations and exerting anti-inflammatory effects (5, 6). ACKR2 is expressed in many parenchymal organs, including barrier tissues like skin, lung, gut and placenta (7). Further analysis revealed that ACKR2 is mainly expressed on lymphatic endothelial cells in resting tissues. Additionally, ACKR2 is expressed by leukocyte populations, including T lymphocytes and their subsets, innate-like B cells, and alveolar macrophages (8–10). Under resting conditions, expression of ACKR2 is primarily found intracellularly. After ligand binding ACKR2 activates a β arrestin-dependent pathway that increases receptor abundance on the cell surface to optimize chemokine uptake and subsequent degradation (11). Next to regulation of ACKR2 mRNA expression levels this suggests a post-transcriptional control of its expression in the cell membrane under inflammatory conditions (8).

ACKR2 was shown to limit inflammatory processes and tissue injury in several disease models, including *Mycobacterium tuberculosis* infection, skin inflammation, cardiac remodeling, and immune complex-mediated glomerulonephritis (12–15). In contrast, in the context of autoimmune disease, ACKR2 expression on lymphatic endothelium reduces accumulation of inflammatory chemokines around lymphatic capillaries, which facilitates fluid flow and migration of activated dendritic cells (DCs) into regional lymph nodes to prime autoreactive T cells (16, 17). Currently, it remains unknown whether ACKR2 could also protect from autoimmune injury such as lupus nephritis, by limiting local chemokine production and inflammation. In contrast, ACKR2, like in other autoimmune diseases (18, 19), may facilitate priming of autoimmune T cells which mediate organ injury.

Given the unknown contribution of ACKR2 to organ damage caused by systemic autoimmune disease like SLE we compared inflammatory injury and systemic autoimmune responses in wildtype (WT) and Ackr2-deficient lupus-prone B6lpr mice to explore potential anti-inflammatory or systemic autoimmunity-augmenting functions of ACKR2.

## Materials and methods

### Animal studies

Ackr2-deficient (Ackr2-/-) mice on the C57BL/6J background were established as previously described (13, 20). After generating Ackr2-deficient B6lpr/lpr (Ackr2-/- B6lpr) mice by crossing Ackr2-deficient with Fas-mutated (lpr/lpr) C57BL/6J mice, the phenotype of female mice at 28 weeks of age was compared to WT B6lpr littermates. Genotyping of individual mice was performed as described (20). All experimental procedures were carried out according to the German animal care and ethics legislation and approved by local government authorities.

### Assessment of autoimmune tissue injury and immunohistochemistry

Plasma creatinine and urea levels were determined using the Jaffe method (Creatinine FS, Diasys, Germany) and enzymatic method (Urea FS, Diasys, Germany), respectively. Albuminuria was determined in spot urine samples by ELISA (Bethyl laboratories, TX, USA) following the manufacturer’s instruction. Organs were harvested following perfusion with phosphate-buffered saline. Kidneys and lungs were embedded in paraffin, and sections were used for periodic acid-Schiff (PAS) staining or immunohistochemistry following routine protocols. The severity of renal lesions was graded using the indices for activity and chronicity as described for human nephritis (21). The severity of lung lesions was scored semi-quantitatively with scores ranging from 0 to 3 by evaluating the extent of perivascular, peribronchial, or interstitial lymphocyte infiltration as described (22). Immunostaining was performed using the following primary antibodies: rat anti-mouse Ly-6B.2 (clone 7/4), rat anti-mouse F4/80 (clone Cl:A3-1), rat anti-human CD3 (clone CD3-12, all from AbD Serotec, Oxford, UK), rat anti-mouse ER-HR3 (BMA Biomedicals, Augst, Switzerland), and mouse anti-mouse α-smooth muscle actin (α-SMA, clone 1A4, DAKO Agilent, Santa Clara, CA, USA). Immunohistochemistry for renal CCL2 and CCL5 was performed with polyclonal rabbit antisera (Invitrogen, Carlsbad, CA, USA). Biotinylated secondary antibodies were detected using an avidin-biotin kit with horseradish peroxidase-based detection (Vector, Burlingame, CA, USA) and 3’3’diaminobenzidine as the substrate. Staining was negative for controls omitting the primary antibody. Glomerular deposition of murine IgG was determined after staining paraffin-embedded sections with biotinylated goat anti-mouse IgG antibody (clone BA-9200, Vector). For quantitative analysis, stained cells were counted in 50 glomeruli or 10 cortical high-power fields per mouse, or were quantified as the fraction of stained area using ImageJ (National Institutes of Health, Bethesda, Maryland, USA). Abundance of CCL2 and CCL5 in the renal interstitium was scored semi-quantitatively with scores ranging from 0 to 3. All assessments were performed in a blinded protocol.

### Analysis of chemokine protein levels by ELISA

Chemokine levels in plasma, total kidney and lung lysates were determined using commercially available ELISA kits for CCL2 and CCL5 (R&D Systems, Abingdon, UK) following the manufacturer’s protocols. The protein content of each kidney and lung sample was determined using Bradford protein assay.

### Autoantibody analysis

Plasma autoantibody levels were measured by ELISA as described (23). For quantification of anti-dsDNA antibodies, NUNC-MaxiSorp ELISA plates (Thermo Fisher Scientific, Schwerte, Germany) were precoated with poly-L-lysine (Trevigen, Gaithersburg, MA, USA) and mouse dsDNA. After incubation with diluted plasma samples, dsDNA-specific IgG, IgG1, IgG2a, IgG2c, IgG3 and total plasma levels of IgG were determined by ELISA (Bethyl laboratories). For anti-histone and anti-Smith antibodies, ELISA plates were precoated with Smith antigen (SMA-3000, Immunovision, Springdale, AR, USA) or histones (10 µg/ml, USB, Cleveland, OH, USA). Plasma levels of rheumatoid factor were determined with a commercial ELISA kit (Fujifilm Wako, Osaka, Japan). Absorbance was measured at 450 nm or 410 nm with a Sun-rise plate reader.

### Flow cytometry for leukocytes in kidney, spleen and lymph nodes

Cell suspensions were prepared and stained with antibodies as described previously (15, 24). Anti-mouse CD3, CD4, CD8, CD19, CD21, CD23, CD25, B220, IgD, IgM, k-light chain, CD138, CD11c, Ly6G, Ly6C (all from BD Pharmingen, Heidelberg, Germany), and F4/80 (AbDSeroTec, Düsseldorf, Germany) were used to identify leukocyte subsets as follows: total leukocytes (CD45+), T lymphocytes (CD45+ CD3+), T helper cells (CD45+ CD3+ CD4+), regulatory T helper cells (CD3+ CD4+ CD25+), cytotoxic T cells (CD45+ CD3+ CD8+), double negative T cells (CD45+ CD3+ CD4- CD8-), myeloid leukocytes (CD45+ CD11b+), neutrophils (CD45+ CD11b+ Ly6G+), inflammatory macrophages (CD45+ CD11b+ CD11c- Ly6C^high^), dendritic cells (CD45+ CD11b+ CD11c+), B lymphocytes (CD45+ B220+ CD19+), mature B cells (B220+ IgM+ IgD+), transitional B cells (B220+ IgM+ IgD-), follicular B cells (B220+, CD21^low^, CD23^high^), marginal zone B cells (B220+, CD21^high^, CD23^low^), and plasma cells (B220+/- CD138+ k-light chain+). Staining for major histocompatibility complex II (MHCII), CD40 (antibodies from BD Pharmingen), and CD69 (Caltag Laboratories, Buckingham, UK) was used to evaluate leukocyte activation. Specific staining of cell subpopulations was demonstrated by respective isotype antibodies. Gating strategies for leukocytes in kidney and spleen or lymph nodes are illustrated in **Supplementary Figures 1** and **2**, respectively. Cell counting beads (Molecular Probes, Eugene, OR, USA) were used for quantification of cells by flow cytometry.

### Quantitative reverse transcriptase polymerase chain reaction

Total RNA was extracted from whole kidneys and lungs using the Purelink RNA Mini Kit (Ambion, Foster City, CA, USA). SYBR Green master mix (Invitrogen) was used for real time PCR on a Light Cycler 480 (Roche, Mannheim, Germany). Gene-specific primers (Metabion, Martinsried, Germany) are listed in **Table 1**. All samples were run in duplicate and normalized to 18S ribosomal RNA expression.

**Table 1:** Primers used for qRT-PCR.

Gene	Forward sequence	Reverse sequence
18s	GCAATTATTCCCCATGAACG	AGGGCCTCACTAAACCATCC
Ackr2	CTTCTTTTACTCCCGCATCG	TATGGGAACCACAGCATGAA
Arg-1	AGAGATTATCGGAGCGCCTT	TTTTTCCAGCAGACCAGCTT
CCL2	CCTGCTGTTCACAGTTGCC	ATTGGGATCATCTTGCTGGT
CCL5	CCACTTCTTCTCTGGGTTGG	GTGCCCACGTCAAGGAGTAT
CCL12	GCTGGACCAGATGCGGTG	CCGGACGTGAATCTTCTGCT
CCR2	GGGCATTGGATTCACCAC	CCGTGGATGAACTGAGGTAA
CXCL10	GGCTGGTCACCTTTCAGAAG	ATGGATGGACAGCAGAGAGC
CTGF	AGCTGACCTGGAGGAAAACA	CCGCAGAACTTAGCCCTGTA
Fizz-1	CCCTTCTCATCTGCATCTCC	CTGGATTGGCAAGAAGTTCC
FSP-1	CAGCACTTCCTCTCTCTTGG	TTTGTGGAAGGTGGACACAA
iNOS	AGGGTCTGGGCCATAGAACT	TGAAGAAAACCCCTTGTGCT
KIM-1	TCAGCTCGGGAATGCACAA	TGGTTGCCTTCCGTGTCTCT
Laminin	CATGTGCTGCCTAAGGATGA	TCAGCTTGTAGGAGATGCCA
MSR-1	CCTCCGTTCAGGAGAAGTTG	TTTCCCAATTCAAAAGCTGA
MRC-1	ATATATAAACAAGAATGGTGGGCAGT	TCCATCCAAATGAATTTCTTATCC
NGAL	AATGTCACCTCCATCCTG	ATTTCCCAGAGTGAACTG
Procollagen 1	ACATGTTCAGCTTTGTGGACC	TAGGCCATTGTGTATGCAGC
Procollagen 4	GTCTGGCTTCTGCTGCTCTT	CACATTTTCCACAGCCAGAG
TNFα	CCACCACGCTCTTCTGTCTAC	AGGGTCTGGGCCATAGAACT
TNFR1	CTTCATTCACGAGCGTTG	ATGGATGTATCCCCATCA
TNFR2	GTCTTCGAACTGCAGCTG	AGATCTGGCACTCGTACC
TGFβ	GGAGAGCCCTGGATACCAAC	CAACCCAGGTCCTTCCTAAA
α-SMA	ACTGGGACGACATGGAAAAG	GTTCAGTGGTGCCTCTGTCA
TLR-7	GTGATGCTGTGTGGTTTGTCTGG	CCTTTGTGTGCTCCTGGACCTA
TLR-9	GCTGTCAATGGCTCTCAGTTCC	CCTGCAACTGTGGTAGCTCACT
IL-1β	TTCCTTGTGCAAGTGTCTGAAG	CACTGTCAAAAGGTGGCATTT
IL-12	GATTCAGACTCCAGGGGACA	GGAGACACCAGCAAAACGAT
Ifit1	CAAGGCAGGTTTCTGAGGAG	GACCTGGTCACCATCAGCAT

### Separation and stimulation of glomeruli and tubulointerstitial tissue

For isolation of glomeruli and tubulointerstitial tissue from 6-8 week-old WT- and Ackr2-/- B6lpr mouse kidneys, a magnetic bead-based separation method originally published by Takemoto et al. (25) was used and performed as previously described. A total of 12,500 glomeruli or 100 µl of tubulointerstitial cell suspensions standardized to a protein content of 0.5 mg/ml were kept in Roswell Park Memorial Institution (RPMI) medium with 15% fetal calf serum (FCS), 15 mM HEPES, 0.66 U/ml insulin and penicillin-streptomycin (PS) at 37°C for 24 hours. Subsequently, tissue was stimulated with 50 ng/ml recombinant murine tumor necrosis factor α (TNFα) or PBS in FCS/PS free RPMI medium for additional 24 hours. CCL2 concentration was determined in supernatants by ELISA.

### Statistical analysis

Data are expressed as mean ± SD as indicated. One-way analysis of variance (ANOVA) followed by Newman-Keult or *post hoc* Tukey test was used for multiple comparisons using GraphPad Prism version 8.01. *P*<0.05 was considered significant.

## Results

### Ackr2-dependent chemokine scavenging in tubulointerstitial tissue of B6lpr kidneys and increased renal Ackr2 expression in B6lpr mice with lupus nephritis

**Figure 1:**
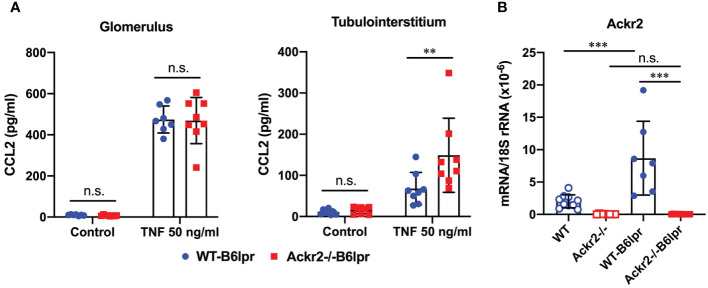
Ackr2 deficiency increases renal chemokine production in tubulointerstitial tissue of Ackr2-/- B6lpr mice *in vitro* and renal Ackr2 mRNA expression is induced in lupus nephritis of B6lpr mice *in vivo*. **(A)** Concentration of CCL2 in unstimulated (control) and TNFα-stimulated glomerular and tubulointerstitial tissue of WT- and Ackr2-/- B6lpr mice is shown. Data represent mean ± SD of 8 mice per genotype, with glomeruli and tubulointerstitial tissue isolated from the same mice. **(B)** mRNA expression levels of Ackr2 were determined in healthy WT and Ackr2-/- control mice, and WT-B6lpr and Ackr2-/- B6lpr mice. Data represent mean ± SD of 7 to 14 mice per group. ***p<0.001; **p<0.01; n.s., not significant.

The potential ability of ACKR2 to limit renal inflammation and injury in lupus nephritis of B6lpr mice was first explored *in vitro* using glomeruli and tubulointerstitial tissue isolated from WT- and Ackr2-/- B6lpr mice. Untreated glomeruli and tubulointerstitial tissue of both genotypes showed minimal and comparable secretion of the pro-inflammatory chemokine CCL2 into supernatants. Inflammatory stimulation with TNFα for 24 hours increased production of CCL2 in the two compartments of both genotypes, with significantly more CCL2 secretion being present in Ackr2-deficient tubulointerstitial cells, but not glomeruli in comparison to WT (**Figure 1A**). These results are consistent with reduced Ackr2-dependent degradation of chemokines in the tubulointerstitial compartment. The restriction of ACKR2 effects to the tubulointerstitial tissue fraction correlates with the known parenchymal expression of renal ACKR2 in the interstitial lymphatic endothelium, which is not present in glomeruli. Furthermore, mRNA expression analysis demonstrated that renal Ackr2 expression was induced by 4.3-fold in female WT-B6lpr mice with lupus nephritis at week 28 of age compared to age-matched healthy B6 WT controls without the lpr mutation. No expression was detected in Ackr2-/- mice (**Figure 1B**). Together, this data suggested a potential anti-inflammatory effect of Ackr2 in lupus nephritis of B6lpr mice by reducing local chemokine production in kidneys.

### Ackr2 deficiency does not affect renal injury in WT-B6lpr mice

**Figure 2:**
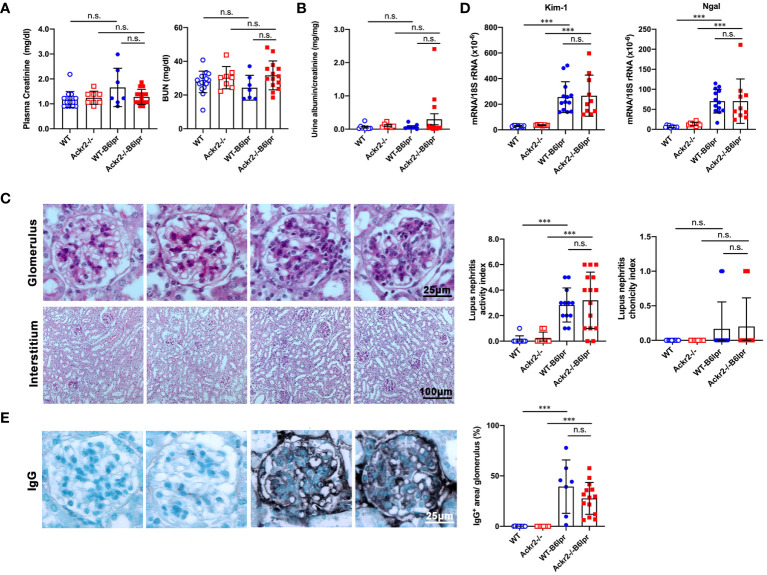
Ackr2 deficiency does not affect renal function and injury in female B6lpr mice at week 28 of lupus nephritis. **(A)** Plasma creatinine and blood urea nitrogen (BUN) levels were determined in healthy WT and Ackr2-/- mice, and WT-B6lpr and Ackr2-/- B6lpr mice at 28 weeks of age. **(B)** Urinary albumin to creatinine ratios (UACR) of mice at 28 weeks of age are shown. **(C)** Staining of renal sections of 28 week old female mice from all groups with periodic acid-Schiff (PAS) reagent demonstrated mesangial hypercellularity in WT- and B6lpr-/- mice. Glomerular injury was comparable in WT- and Ackr2-/- B6lpr mice, as demonstrated by similar activity and chronicity indices. **(D)** Kidney mRNA expression of the tubular injury markers Kim-1 and Ngal as measured by real-time quantitative PCR. **(E)** Renal sections were stained for deposited murine IgG. Representative images of glomeruli (original magnification x400) and interstitium (original magnification x200) from healthy WT- and Ackr2-/- control mice, and WT- and Ackr2-/-B6lpr mice are shown. Semiquantitative and morphometric analysis was performed as described in the Materials and methods. Data represent mean ± SD of 7 to 15 mice per group. ***p<0.001; n.s., not significant.

To characterize a potential nephroprotective effect of Ackr2 in B6lpr mice with lupus nephritis we first evaluated excretory kidney function in female WT- and Ackr2-/- B6lpr mice at the age of 28 weeks. Plasma creatinine and blood urea nitrogen (BUN) levels did not significantly increase compared to age-matched healthy WT- und Ackr2-deficient mice without the lpr mutation and were comparable between both genotypes (**Figure 2A**). Similarly, albuminuria as a functional marker of glomerular injury did not increase in both WT- and Ackr2-/- B6lpr mice until week 28 (**Figure 2B**). Despite unaffected renal functional parameters, WT- and Ackr2-deficient B6lpr mice had developed diffuse proliferative glomerulonephritis by 28 weeks of age, with the presence of mesangial cell proliferation and glomerular hypercellularity as revealed by histology of PAS-stained kidney sections (**Figure 2C**). However, the glomerular injury was comparable in WT- and Ackr2-/- B6lpr mice, as demonstrated by similar activity and chronicity indices for lupus nephritis after morphometric assessment (**Figure 2C**). Moreover, WT- and Ackr2-/- B6lpr mice revealed increased renal mRNA expression of the tubular injury markers Kim-1 and Ngal compared to healthy WT and Ackr2-/- controls, without differences between the two genotypes (**Figure 2D**). Glomerular deposition of murine IgG was not significantly different in B6lpr mice of both genotypes while almost no IgG deposition was detected in healthy WT and Ackr2-/- control mice (**Figure 2E**). Thus, B6lpr mice developed lupus nephritis with mild glomerular and tubular injury until week 28, but impaired tubulointerstitial chemokine scavenging in Ackr2-deficient mice did not result in more severe renal injury.

### Ackr2 deficiency increases tubulointerstitial T and B cell infiltration in B6lpr mice

**Figure 3:**
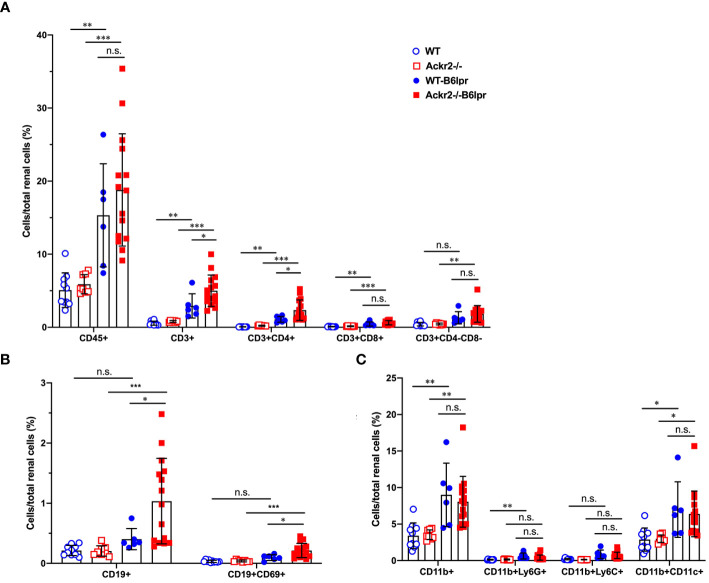
Ackr2 deficiency increases renal accumulation of CD4+ T cells and B cells in female B6lpr mice at week 28 of lupus nephritis. Flow cytometry was applied to quantify **(A)** total leukocyte numbers and T cell subsets, **(B)** B cells, **(C)** CD11b+ mononuclear phagocytes, CD11b+ Ly6G+ granulocytes, CD11b+ Ly6C^high^ inflammatory macrophages, and CD11b+ CD11c+ dendritic cells (DCs) in WT- and Ackr2-/- control mice, and WT- and Ackr2-/- B6lpr mice. Data represent mean ± SD of 6 to 15 mice per group. *p<0.05; **p<0.01; ***p<0.001; n.s., not significant.

To investigate whether Ackr2-deficiency with impaired chemokine clearance would correlate with increased renal inflammatory cell infiltrates in B6lpr mice, renal leukocyte subsets were quantified by flow cytometry in kidneys at week 28. Flow cytometry revealed an increase of investigated leukocyte subsets in B6lpr mice of both genotypes compared to healthy controls without the lpr mutation. Moreover, significantly increased numbers of CD3+ CD4+ T cells and CD19+ B cells, including the activated CD69+ CD19+ B cell subset were present in kidneys of Ackr2-deficient B6lpr mice compared to the WT-B6lpr group. In contrast, numbers in WT- and Ackr2-/- B6lpr mice were not different for CD3+ CD8+ T cells, CD3+ CD4- CD8- autoreactive T cells, CD11b+ Ly6G+ neutrophils, CD11b+ mononuclear phagocytes, CD11b+ Ly6C^high^ inflammatory macrophages, and CD11b+ CD11c+ DCs (**Figure 3**).

**Figure 4:**
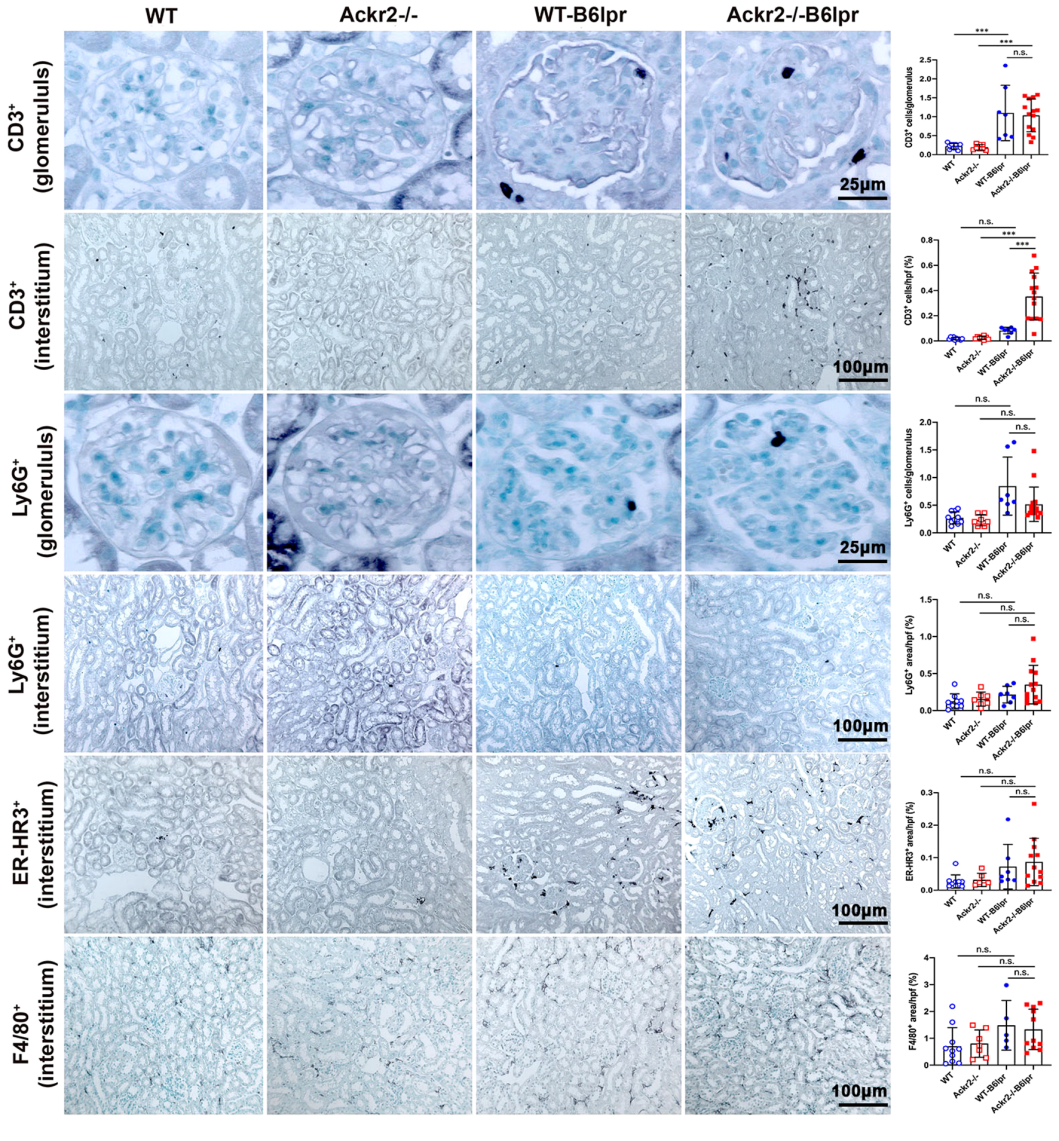
Compartment-specific analysis of renal leukocyte infiltration in female B6lpr mice at week 28 of lupus nephritis. Renal sections of WT and Ackr2-/- control mice, and WT- and Ackr2-/- B6lpr mice were stained for CD3 (lymphocytes), Ly6G (granulocytes), and ER-HR3 and F4/80+ (macrophages). Representative images of glomeruli (original magnification x400) and interstitium (original magnification x200) are shown. Morphometric analysis was performed as described in the Materials and methods. Data represent mean ± SD of 7 to 14 mice per group. ***p<0.001; n.s., not significant.

Consistently, immunohistochemistry, which allowed for additional compartment-specific evaluation of renal leukocyte infiltrates, revealed significantly increased numbers of tubulointerstitial, but not glomerular CD3+ T cells in Ackr2-/- B6lpr compared to WT B6lpr mice. Numbers of glomerular and tubulointerstitial Ly6G+ neutrophils, as well as tubulointerstitial ER-HR3+ and F4/80+ macrophages were comparable between B6lpr mice of the two genotypes (**Figure 4**). Thus, Ackr2 deficiency in B6lpr mice resulted in increased tubulointerstitial T and B cell infiltration, but did not affect neutrophil or macrophage accumulation in kidneys with lupus nephritis. Expansion of this tertiary lymphoid tissue, however, was not associated with more severe renal injury in Ackr2-/- B6lpr mice.

### Ackr2 deficiency does not affect renal inflammation in B6lpr mice

**Figure 5:**
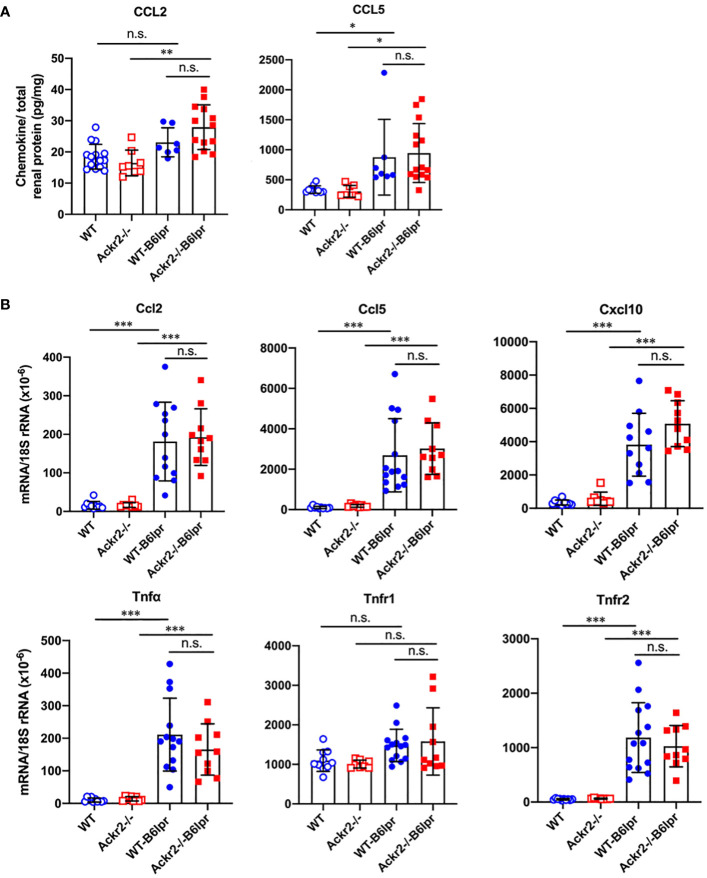
Renal CCL2 and CCL5 protein content and renal mRNA expression of inflammatory markers in female B6lpr mice at week 28 of lupus nephritis. **(A)** Renal protein levels of CCL2 and CCL5 were determined in WT and Ackr2-/- control mice, and WT- and Ackr2-/-B6lpr mice. **(B)** Renal mRNA expression of pro-inflammatory chemokines Ccl2, Ccl5, Cxcl10, the cytokine Tnfα and its receptors Tnfr1 and Tnfr2 in WT and Ackr2-/- control mice, and WT- and Ackr2-/- B6lpr mice. TNF, tumor necrosis factor. Data represent mean ± SD of 7 to 14 mice per group. *p<0.05; **p<0.01; ***p<0.001; n.s., not significant.

To analyze whether increased renal lymphocyte infiltrates in Ackr2-deficient B6lpr mice would correlate with more severe renal inflammation, renal expression of inflammatory mediators was investigated. By ELISA, increased renal protein levels of the proinflammatory chemokines CCL2 and CCL5 could be detected in B6lpr mice of both genotypes compared to healthy B6 controls, but these were similar in respective WT- and Ackr2-/- kidneys (**Figure 5A**). These findings were confirmed by immunohistochemistry for CCL2 and CCL5, which demonstrated an increased abundance of both chemokines in the glomerular and interstitial renal compartment of B6lpr mice compared to healthy controls, with comparable expression levels in WT- and Ackr2-/-B6lpr kidneys (**Supplementary Figure 3**). The mRNA expression of inflammatory chemokines, TNFα and its receptors was comparable in healthy WT and Ackr2-/- B6 control mice and increased in B6lpr mice of both genotypes with lupus nephritis at week 28. However, all analyzed inflammatory mediators were similarly expressed in WT- and Ackr2-/- B6lpr mice with lupus nephritis (**Figure 5B**).

These results indicate that, together with similar numbers of renal granulocytes, macrophages and DCs, Ackr2 deficiency did not augment kidney inflammation in B6lpr mice with lupus nephritis, despite increased renal lymphocyte accumulation. Moreover, in contrast to our *in vitro* data demonstrating impaired chemokine clearance by Ackr2-deficient tubulointerstitial tissue, absence of Ackr2-dependent chemokine scavenging did not increase renal chemokine levels in B6lpr mice *in vivo*. Apparently, reduced Ackr2-mediated chemokine degradation can be compensated by alternative mechanisms in Ackr2-deficient mice *in vivo*.

### Ackr2 deficiency does not affect renal fibrosis in B6lpr mice

**Figure 6:**
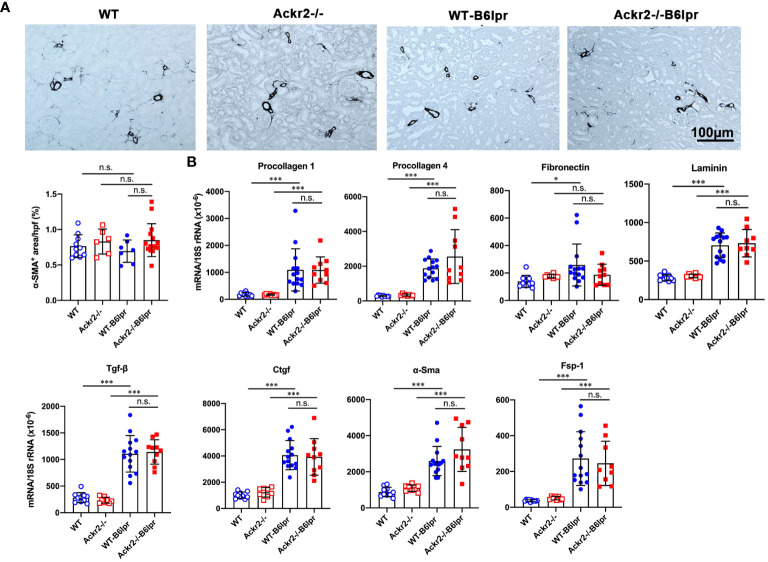
Ackr2 deficiency does not affect markers of renal fibrosis in female B6lpr mice at week 28 of lupus nephritis. **(A)** Renal accumulation of myofibroblasts was analyzed by α-SMA staining. Representative images of renal sections from WT and Ackr2-/- control mice, and WT- and Ackr2-/- B6lpr mice are shown; original magnification x200. Morphometric quantification of α-SMA staining was performed by assessing the ratio of α-SMA positive area per high power field (hpf). **(B)** Renal mRNA expression of the fibrosis-associated markers procollagen 1, procollagen 4, fibronectin, laminin, Tgf-β, Ctgf, α-Sma, and Fsp-1 was analyzed in WT and Ackr2-/- healthy control and B6lpr mice. Tgf-β, transferring growth factor; Ctgf, connective tissue growth factor; α-Sma, α-smooth muscle actin; Fsp-1, fibroblast-specific protein 1. Data represent mean ± SD of 7 to 14 mice per group. *p<0.05; ***p<0.001; n.s., not significant.

Persistent renal inflammation leads to renal fibrosis, which is an important prognostic marker for development of chronic renal functional impairment in progressive kidney disease including lupus nephritis. α-SMA+ myofibroblasts contribute to tissue fibrosis. Therefore, α-SMA staining was used to analyze the extent of kidney fibrosis. As shown in **Figure 6A**, in addition to physiological staining of arterial vessel walls α-SMA+ fibroblasts were mainly located in the tubulointerstitium. However, α-SMA staining was comparable between healthy control and B6lpr mice and was not different in WT and Ackr2-deficient groups. In contrast, compared to healthy WT and Ackr2-/- controls renal mRNA expression of extracellular matrix components and markers of fibrotic remodeling including procollagen 1, procollagen 4, fibronectin, laminin, transforming growth factor-β (Tgf-β), connective tissue growth factor (Ctgf), α-Sma and the fibroblast marker fibroblast-specific protein 1 (Fsp-1) significantly increased in WT- and Ackr2-/- B6lpr mice, but was not different between the two genotypes (**Figure 6B**). Therefore, Ackr2 deficiency did not affect the extent of renal fibrosis in B6lpr mice with lupus nephritis at week 28, being consistent with comparable renal inflammation in WT- and Ackr2-/- B6lpr mice.

### Ackr2 deficiency aggravates peribronchial T cell infiltration in B6lpr mice

**Figure 7:**
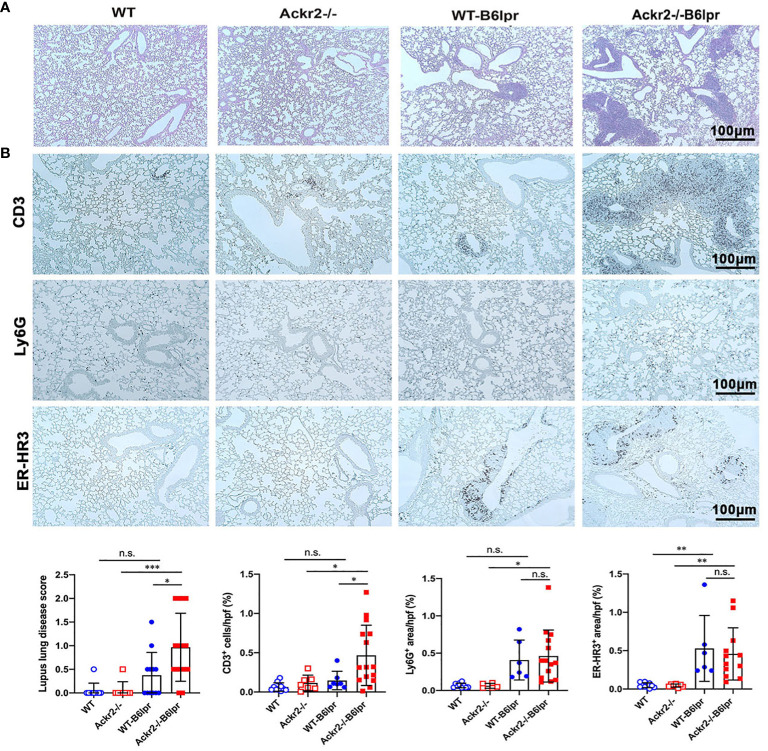
Effect of Ackr2 deficiency on autoimmune lung disease in female B6lpr mice at week 28. **(A)** Representative images of PAS-stained lung sections from WT and Ackr2-/- control mice, and WT- and Ackr2-/- B6lpr mice at 28 weeks of age are shown; original magnification x200. **(B)** Lung sections of WT and Ackr2-/- control mice, and WT- and Ackr2-/- B6lpr mice were stained with antibodies for CD3 (lymphocytes), Ly6G (granulocytes), and ER-HR3 (macrophages), original magnification x200. Semiquantitative and morphometric analysis was performed as described in the Materials and Methods. PAS, periodic acid-Schiff. Data represent mean ± SD of 6 to 15 mice per group. *p<0.05; **p<0.01; ***p<0.001; n.s., not significant.

Lung injury is an additional manifestation in SLE. B6lpr mice developed significant autoimmune lung disease at week 28 of age, with marked peribronchial leukocyte infiltration. Semiquantitative assessment of PAS-stained tissue sections demonstrated a significantly worsened lung injury score based on the extent of peribronchial cell infiltrates. The score was 0.97 ± 0.72 in Ackr2-/- B6lpr mice compared to 0.38 ± 0.48 in WT-B6lpr littermates. Lung pathology was absent in age-matched healthy WT and Ackr2-/- control mice (**Figure 7A**). Immunohistochemistry revealed significantly increased numbers of peribronchial and perivascular CD3 T cell infiltrates in Ackr2-/- B6lpr mice, forming tertiary lymphoid tissue. In contrast, numbers of pulmonary granulocytes and ER-HR3 positive macrophages were comparable to WT-B6lpr mice (**Figure 7B**). Of note, next to peribronchal T cell infiltrates no other parenchymal lung injury, e.g. alveolitis or interstitial fibrosis could be detected in WT- or Ackr2-/- B6lpr mice.

**Figure 8:**
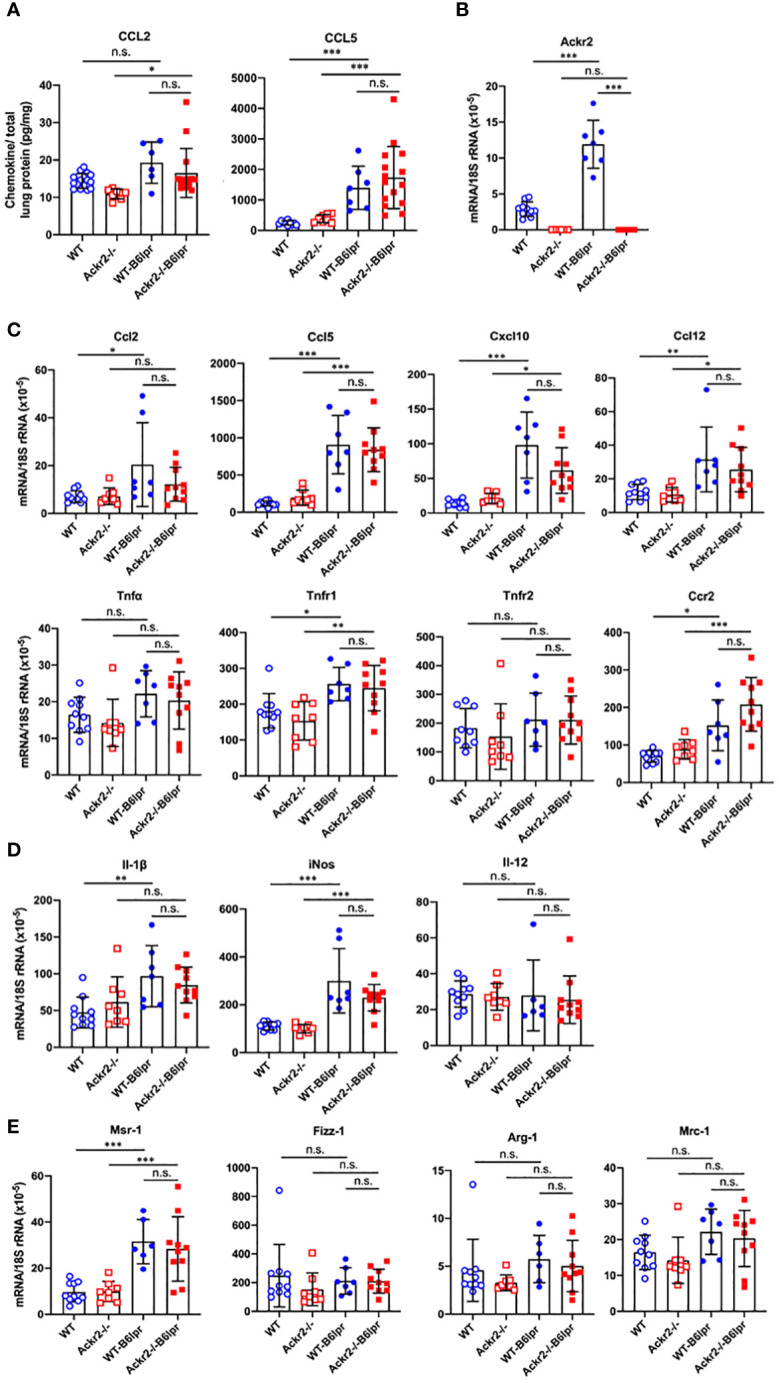
Pulmonary chemokine levels and mRNA expression of inflammatory mediators in female B6lpr mice at week 28. **(A)** Lung protein levels of CCL2 and CCL5 were determined in WT and Ackr2-/- control mice, and WT- and Ackr2-/- B6lpr mice. **(B)** Pulmonary Ackr2 mRNA expression in WT-B6lpr mice at week 28 was induced compared to healthy WT controls. **(C)** Pulmonary mRNA expression levels of pro-inflammatory chemokines Ccl2, Ccl5, Ccl12, Cxcl10, chemokine receptor Ccr2, Tnfα and its receptors Tnfr1 and Tnfr2 in WT and Ackr2-/- control mice, and WT- and Ackr2-/- B6lpr mice are shown. **(D)** Pulmonary mRNA expression of M1 and **(E)** M2 macrophage markers was analyzed in WT and Ackr2-/- control mice, and WT- and Ackr2-/-B6lpr mice. Il, interleukin; iNos, inducible nitric oxide synthase; Mrc, mannose receptor; Fizz-1, resistin-like molecule α1; Arg, arginase; Msr, macrophage scavenger receptor. Data represent mean ± SD of 6 to 15 **(A)** and 7-10 **(B, C)** mice per group. *p<0.05; **p<0.01; ***p<0.001; n.s., not significant.

As increased pulmonary T cell recruitment in Ackr2-deficient B6lpr mice may be mediated by reduced Ackr2-dependent chemokine scavenging and aggravated inflammation, the extent of pulmonary inflammation in WT- and Ackr2-/- B6lpr mice was compared. Protein levels of CCL2 and CCL5 chemokines increased in lung tissue of B6lpr mice compared to healthy controls, but were not significantly different between the two genotypes (**Figure 8A**). This was despite a significantly induced pulmonary mRNA expression of Ackr2 in WT-B6lpr mice compared to healthy WT controls (**Figure 8B**), which suggested an increase in Ackr2-dependent chemokine scavenging activity in lung tissue of B6lpr mice. Moreover, mRNA expression of the chemokines Ccl2, Ccl5, Ccl12, Cxcl10, the Ccl2 receptor Ccr2, inflammatory cytokines like TNFα and its receptors (**Figure 8C**) as well as M1 and M2 macrophage markers (**Figures 8D, E**) were comparable between WT- and Ackr2-/- B6lpr mice. Thus, Ackr2 deficiency aggravated peribronchial accumulation of CD3+ T cells in B6lpr mice, without significantly affecting numbers of granulocytes, macrophages, and inflammatory activity in lungs of Ackr2-/- mice. Moreover, like in the kidney, lack of Ackr2-mediated chemokine scavenging did not result in increased pro-inflammatory chemokine levels in lung tissue, suggesting compensatory mechanisms for local chemokine clearance in Ackr2-deficient B6lpr mice.

### Ackr2 deficiency does not affect autoantibody levels and systemic B cell expansion in B6lpr mice

**Figure 9:**
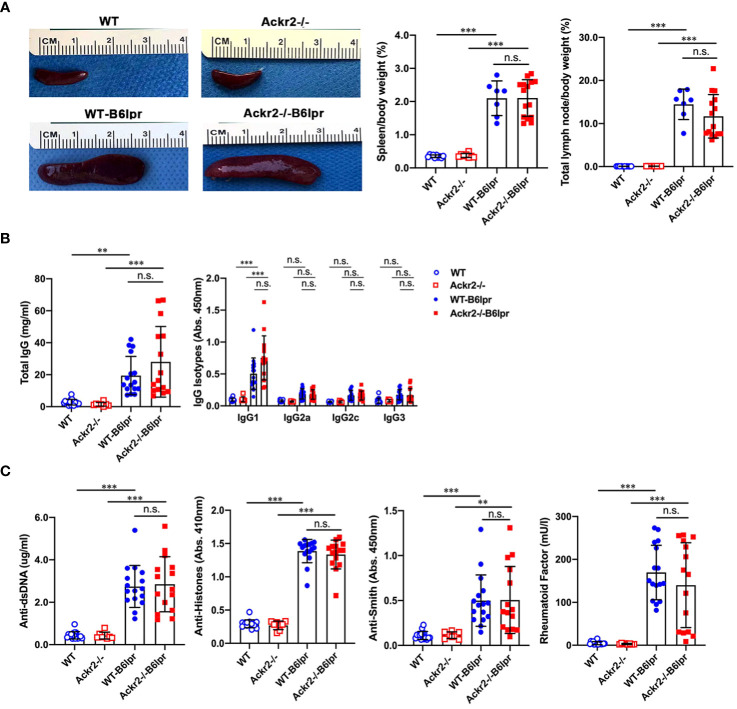
Effects of Ackr2 deficiency on lymphoproliferation and autoantibody production in female B6lpr mice at week 28. **(A)** Compared to WT and Ackr2-/- control mice, WT- and Ackr2-/- B6lpr mice showed similarly increased spleen and total lymph node weights. Representative images of spleens of all groups at 28 weeks of age are shown. **(B)** Plasma levels of total IgG, IgG isotypes and **(C)** lupus autoantibodies including dsDNA autoantibodies, anti-histone antibodies, anti-Smith antibody, and rheumatoid factor were measured by ELISA revealing similarly increased levels in B6lpr mice of both genotypes at 28 weeks. Data represent mean ± SD of 7 to 15 mice per group. **p<0.01; ***p<0.001; n.s., not significant.

Next, potential effects of Ackr2 deficiency on systemic autoimmune activity were investigated. Female WT- and Ackr2-/- B6lpr mice developed a lymphoproliferative syndrome evident from spleen and total lymph node weights. However, spleen weight and total lymph node weights were comparable in WT- and Ackr2- deficient B6lpr mice (**Figure 9A**). We next evaluated the effect of the Ackr2 genotype on lupus autoantibody production. At 28 weeks of age, plasma levels of total IgG and IgG1 significantly increased in female WT- and Ackr2-/- B6lpr mice compared to their respective healthy WT and Ackr2-/- controls. However, total IgG and all IgG isotype levels were comparable in WT- and Ackr2-/- B6lpr mice, demonstrating unaltered humoral immune activity in Ackr2- deficient B6lpr mice (**Figure 9B**). With regards to specific anti-nuclear autoantibodies, plasma levels of dsDNA autoantibodies, anti-histone antibodies, rheumatoid factor and Smith autoantibodies increased in B6lpr mice but were also comparable between WT- and Ackr2-/- B6lpr mice (**Figure 9C**).

**Figure 10:**
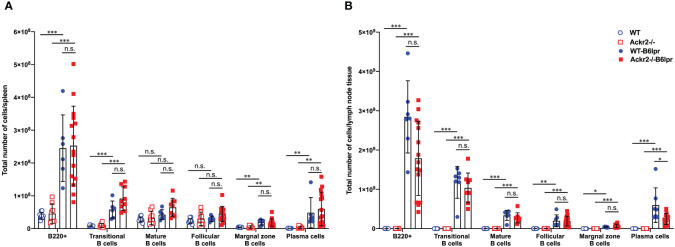
Effect of Ackr2 deficiency on systemic B cell expansion in female B6lpr mice at week 28. Absolute numbers of **(A)** spleen and **(B)** lymph node B cell subsets were quantitated by flow cytometry identifying B220+ IgM+ IgD- transitional B cells, B220+ IgM+ IgD+ mature B cells, B220+ CD21^high^ CD23^low^ marginal zone B cells, B220+ CD21^low^ CD23^high^ follicular B cells, and B220+ CD138+ κ-light chain+ plasma cells and revealed similar numbers in WT- and Ackr2-/- B6lpr mice. Data represent mean ± SD of 7 to 15 mice per group. *p<0.05; **p<0.01; ***p<0.001; n.s., not significant.

Consistent with similar levels of circulating lupus autoantibodies, flow cytometry did not reveal any difference in total numbers of various B cell subsets and plasma cells in spleens of WT- and Ackr2-/- B6lpr mice, although most of these cells were increased in both B6lpr genotypes compared to healthy controls (**Figure 10A**). Similar results were obtained when the total numbers of B cell subsets and plasma cells were determined in lymph node tissue, with the exception of significantly lower numbers of plasma cells in lymph nodes of Ackr2-/- B6lpr mice (**Figure 10B**). Moreover, relative abundances of B cell subsets and plasma cells were also comparable in spleen and lymph nodes of WT- and Ackr2-/- B6lpr mice (**Supplementary Figure 4**). Thus, Ackr2-deficiency in B6lpr mice did not substantially alter systemic humoral autoimmune responses indicated by comparable SLE autoantibody levels and systemic B cell numbers in WT- and Ackr2-/- B6lpr mice.

### Ackr2 deficiency promoted CD4+ T cell expansion in B6lpr mice

**Figure 11:**
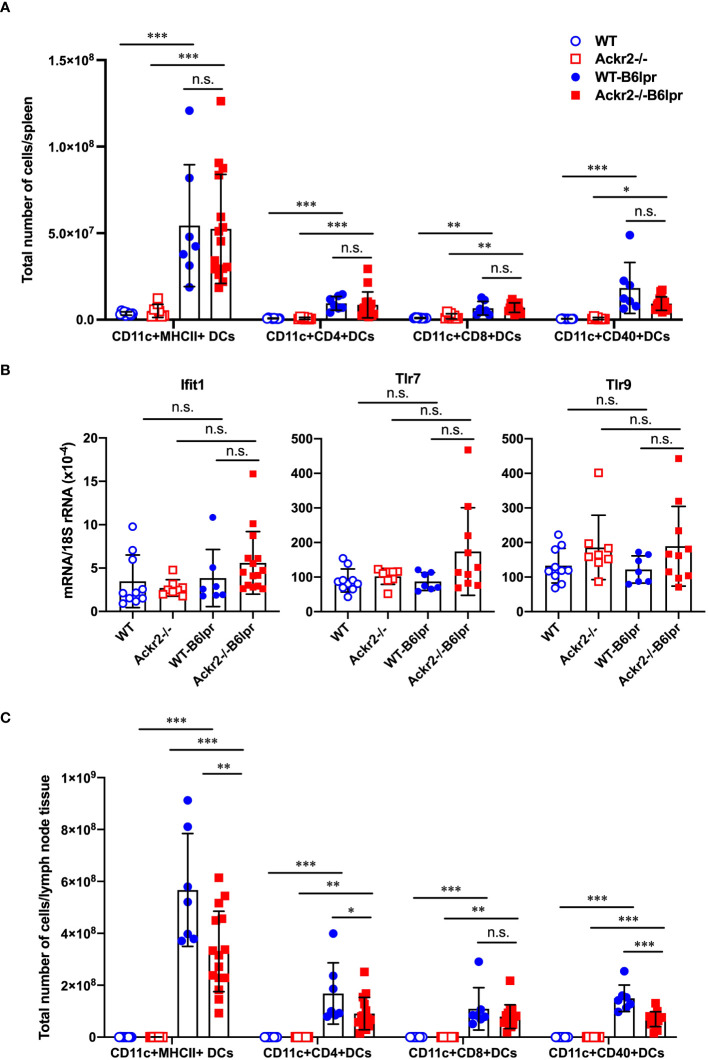
Effect of Ackr2 deficiency on dendritic cells (DCs) in spleens and lymph nodes of female B6lpr mice at week 28. **(A)** Absolute numbers of DC subsets in spleen of WT and Ackr2-/- control mice, and WT- and Ackr2-/- B6lpr mice were quantified by flow cytometry demonstrating similar numbers in B6lpr mice of both genotypes. **(B)** mRNA expression levels of the IFNα-dependent genes Ifit1, Tlr7, and Tlr9 in splenocytes were determined by real-time quantitative PCR. **(C)** Analysis of DC subsets in lymph nodes revealed reduced absolute numbers of CD11c+ CD4+ and activated CD11c+ CD40+ DC subsets in Ackr2-/- B6lpr mice compared to WT B6lpr littermates. MHCII, major histocompatibility complex II; Ifit, interferon-induced protein with tetratricopeptide repeats; TLR, toll like receptor. Data represent mean ± SD of 7 to 15 mice per group. *p<0.05; **p<0.01; ***p<0.001; n.s., not significant.

ACKR2 has been described to facilitate the migration of antigen-presenting cells into draining lymph nodes for effective T cell priming by reducing pro-inflammatory chemokine concentrations in draining lymphatic capillaries (16). We therefore quantified DC subsets and characterized their activation state in spleens and lymph nodes harvested from female WT and Ackr2-/- control mice, as well as female WT- and Ackr2-/- B6lpr mice at 28 weeks of age. Total numbers of DCs and several DC subsets were significantly higher in WT- and Ackr2-deficient B6lpr spleens compared to respective healthy control mice, but were not significantly different between the B6lpr groups (**Figure 11A**). Moreover, DC numbers were also similar in WT- and Ackr2-/- B6lpr spleens when their relative abundance was compared (**Supplementary Figure 5A**). Consistent with this finding mRNA expression of the DC-related IFN-dependent genes Ifit1, Tlr7 and Tlr9 were comparable in spleens of both genotypes (**Figure 11B**). In lymph nodes total and relative DC numbers increased in B6lpr mice of both genotypes compared to healthy WT and Ackr2-/- controls. Interestingly, Ackr2-/- B6lpr mice showed significantly lower absolute, but not relative numbers of CD11c+ MHCII+ DCs, CD11c+ CD4+ DCs, and CD11c+ CD40+ activated DCs in lymph node tissue when compared to WT-B6lpr littermates (**Figure 11C**, **Supplementary Figure 5B**). Analysis of macrophage abundance revealed that numbers of both CD11b+ myeloid leukocytes and CD11b+ F4/80+ MHCII+ macrophages were elevated in spleens and lymph nodes of WT- and Ackr2-/- B6lpr mice compared with healthy B6 controls, but were comparable in the two B6lpr groups (data not shown).

**Figure 12:**
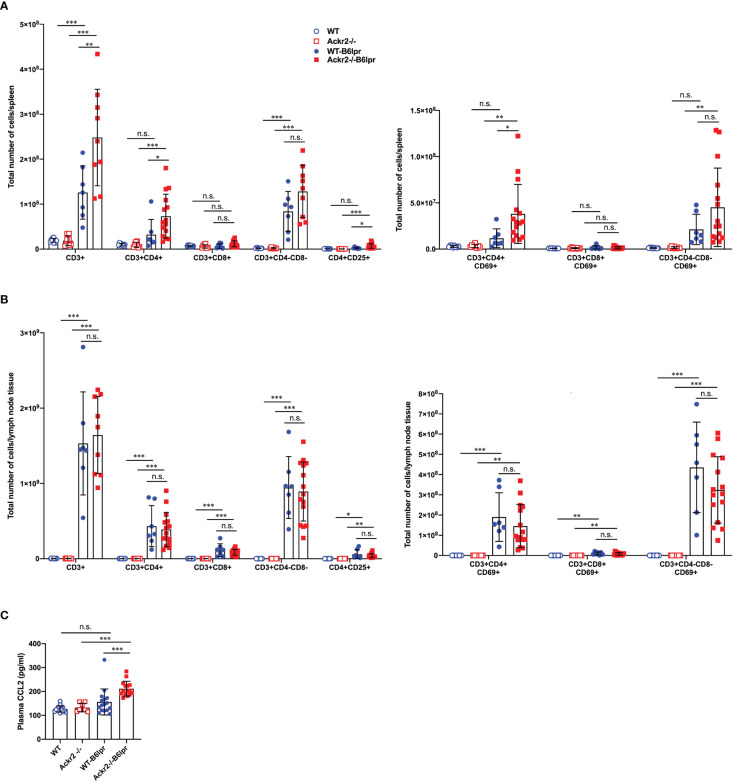
Effect of Ackr2 deficiency on T cell numbers and activation in spleen and lymph nodes, and plasma CCL2 levels in female B6lpr mice at week 28. Absolute numbers of T cell subsets and CD69+ activated T cells were quantified by flow cytometry of **(A)** spleens and **(B)** lymph nodes isolated from WT and Ackr2-/- control mice, and WT- and Ackr2-/- B6lpr mice. Compared to WT-B6lpr mice Ackr2-/- B6lpr mice showed significantly increased numbers of CD3+CD4+ T cells, activated CD69+ CD3+ CD4+ T cells, and CD4+ CD25+ regulatory T cells in spleens, with comparable numbers in lymph nodes. **(C)** Plasma CCL2 levels were determined in WT and Ackr2-/- control mice, and WT- and Ackr2-/- B6lpr mice at 28 weeks of age. Higher CCL2 levels were present in Ackr2-/- B6lpr mice compared to WT B6lpr. Data represent mean ± SD of 7 to 15 mice per group. *p<0.05; **p<0.01; ***p<0.001; n.s., not significant.

To further investigate whether reduced DC numbers and activation in lymph nodes of Ackr2-/- B6lpr mice affected systemic activation of T cells, various T cell subsets were quantified in spleens and lymph nodes of mice at 28 weeks of age by flow cytometry. Our results indicate that absolute numbers of CD3+CD4+ T cells, activated CD69+ CD3+CD4+ T cells, and CD4+CD25+ regulatory T cells in spleens significantly increased in Ackr2-/- B6lpr mice compared to WT-B6lpr littermates, whereas absolute T cell numbers in spleens of WT and Ackr2-/- control mice were low. In contrast, numbers of CD3+CD8+ T cells and autoreactive CD3+CD4-CD8- T cells were similar in WT- and Ackr2-/- B6lpr spleens (**Figure 12A**). Similar results were obtained when the relative abundance of leukocyte subpopulations was analyzed. We found a relative expansion of CD3+ T cells in spleen, with significant increases of CD3+CD4+ T cells and activated CD69+CD3+CD4+ T cells in Ackr2-deficient B6lpr mice compared to WT-B6lpr (**Supplementary Figure 6A**). Moreover, despite reduced accumulation of activated DCs in Ackr2-/- B6lpr lymph nodes absolute T cell numbers and activation in lymph nodes were comparable between WT- and Ackr2-/- B6lpr mice (**Figure 12B**), with a significantly increased relative expansion of CD3+ T cells and double negative T cells in Ackr2-deficient B6lpr mice (**Supplementary Figure 6B**).

Mechanisms of CD4+ T cell expansion in spleens of Ackr2-deficient Bl6lpr mice are not known. However, CCR2-deficiency or blockade sequesters T cells in bone marrow, with concomitant reduction in spleen (26). Conversely, increased levels of CCR2 ligands like CCL2 may facilitate T cell egress from the bone marrow and accumulation in spleen. We therefore measured CCL2 plasma levels. This revealed a trend towards higher plasma CCL2 concentrations in WT-B6lpr mice when compared to control WT mice, and significantly higher CCL2 levels in Ackr2-/- B6lpr mice compared to healthy Ackr2-/- controls. Importantly, plasma CCL2 levels were indeed significantly increased in Ackr2-deficient B6lpr mice compared to the WT-B6lpr group (**Figure 12C**).

In summary, Ackr2 deficiency promoted CD4+ T cell expansion including regulatory T cell subsets in spleens of B6lpr mice. This may be facilitated by increased systemic CCL2 levels, and correlated with enhanced T cell accumulation in affected organs of Ackr2-/- B6lpr mice like kidney and lung. Moreover, reduced absolute but not relative numbers of activated DCs in lymph nodes of Ackr2-deficient B6lpr mice did not translate into reduced T cell activation and systemic autoimmune reactivity.

## Discussion

ACKR2 was described as a chemokine scavenger which limits inflammatory processes and leads to the resolution of inflammation in several disease models including progressive kidney disease (15, 27). However, ACKR2 may also facilitate autoimmune disease such as experimental autoimmune encephalitis by allowing efficient priming of autoreactive T cells in lymphoid organs enhancing encephalitogenic responses (18). It was postulated that ACKR2 expression by lymphatic endothelial cells reduces local chemokine concentrations which allows migration of activated DCs into regional lymph nodes for subsequent T cell activation (16). Conversely, ACKR2-facilitated migration of tissue-resident DCs carrying local antigens to draining lymph nodes to induce tolerance may again protect from autoimmunity and inflammation (28). Since all of these functions play a critical role in the pathogenesis of autoimmune diseases and resulting tissue damage, ACKR2 may have decisive effects on both systemic autoimmunity and autoimmune organ injury. We therefore investigated potential functions of ACKR2 in B6lpr mice modelling SLE and lupus nephritis as a prototypic autoimmune disorder.

Our *in vitro* data demonstrated that Ackr2-deficient tubulointerstitial renal tissue of B6lpr mice has less capacity to down-regulate local chemokine levels upon inflammatory stimulation. Moreover, compared to healthy controls WT-B6lpr mice showed induced mRNA expression of Ackr2 in diseased kidneys and lungs. Thus, we hypothesized that Ackr2 deficiency might enhance renal leukocyte recruitment into inflamed kidneys and lungs of B6lpr mice with lupus nephritis, leading to more severe organ inflammation and injury. Indeed, analysis by flow cytometry revealed increased accumulation of CD4+ T cells and B cells in Ackr2-deficient kidneys of B6lpr mice, but no increase of renal granulocytes, macrophages or DCs. Increased renal T cell infiltration in Ackr2-/- B6lpr mice was confirmed by immunochemistry, which localized it to the interstitium but not glomeruli. This data would be consistent with a reduced ability of Ackr2-deficient tubulointerstitial tissue for chemokine scavenging in inflamed kidneys with lupus nephritis, most likely due to absent Ackr2 expression in interstitial lymphatic endothelium (15). Similarly, peribronchial T cell, but not granulocyte or macrophage infiltration significantly increased in Ackr2-deficient B6lpr mice in comparison to the WT-B6lpr group. However, despite enhanced T cell infiltration into injured SLE kidneys of Ackr2-deficient B6lpr mice, renal injury, numbers of leukocyte effector cells like granulocytes and macrophages, renal expression of inflammatory mediators and markers of fibrotic tissue remodeling were comparable to WT-B6lpr mice. Similar findings were present in pulmonary tissue. Moreover, tissue levels of pro-inflammatory chemokines like the Ackr2 ligand CCL2 were not increased in diseased kidneys and lungs of Ackr2-/- B6lpr mice compared to WT B6lpr littermates. This suggests a redundant role of ACKR2 in controlling local chemokine levels in this SLE model. Apparently, loss of the chemokine scavenging activity of Ackr2 in injured organs could be compensated by alternative mechanisms. Indeed, binding of pro-inflammatory chemokines to their canonical receptors may not only activate target cells but also leads to internalization of bound ligands. For example, some cells like Ly6C^high^ monocytes can express both ACKR2 and CCR2, which results in a strong CCL2 scavenging activity *in vitro* and *in vivo*, with CCR2 scavenging CCL2 much more effectively than ACKR2 (29) Cons.

Systemic cellular and humoral autoimmune activity was not substantially altered in Ackr2-deficient B6lpr mice. Thus, the extent of splenomegaly and lymphadenopathy was comparable between female WT- and Ackr2-/- B6lpr mice, as were numbers of CD3+CD4-CD8- autoreactive T cells in spleen, lymph nodes and kidneys, levels of circulating lupus autoantibodies and the extent of glomerular IgG deposition. Consistently, Ackr2 deficiency did not affect numbers of effector leukocyte subsets like neutrophils and macrophages systemically in spleen and lymph nodes, as it was the case in injured kidneys and lungs. However, distinct effects of Ackr2 deficiency could be detected. Ackr2-/- B6lpr mice demonstrated increased expansion of splenic CD4+ T cells, including regulatory CD4 T cell subsets, and reduced accumulation of activated DCs in lymph nodes. These pro- and anti-inflammatory effects, however, may balance each other leading to a grossly unaffected systemic autoimmune response in Ackr2-/- B6lpr mice. Despite this, lymphocyte infiltrates including CD4+ T cells and B cells were increased in Ackr2-/- B6lpr kidneys, as were peribronchial T cell aggregates in lungs. With unaltered tissue levels of chemokines, this expansion of tertiary lymphoid tissue could be mediated by increased plasma levels of chemokines. Elevated plasma CCL2 levels were present in Ackr2-/- B6lpr mice, and have been reported in Ackr2-deficient mice in other studies (10, 15, 27), These may facilitate mobilization of leukocytes including T cells from the bone marrow into the circulation and peripheral tissue (26). An alternative mechanism promoting tissue infiltration of lymphocytes in Ackr2-deficient B6lpr mice may be the loss of pro-inflammatory chemokine scavenging by cell-autonomous lymphocyte-expressed Ackr2 that would facilitate their activation by local chemokines (8, 9). Active Ackr2 may be less prominently expressed on the cell surface of neutrophils, macrophages and DCs, which may explain unaltered organ infiltration of these leukocyte subtypes into organs of Ackr2-deficient B6lpr mice. Of note, homeostatic chemokines such as CXCL12, CXCL13, CCL19, and CCL21, all of which recruit lymphocytes and are implicated in the formation of tertiary lymphoid tissue (30), are no ACKR2 ligands (6). Thus, Ackr2-deficiency should not increase local homeostatic chemokine levels. Together, sufficient autoimmune responses with increased pro-inflammatory chemokine levels in the circulation, but preserved chemokine scavenging activity in kidneys and lungs of Ackr2-deficient B6lpr mice may lead to increased lymphocyte infiltration into these organs forming tertiary lymphoid structures. However, this did not affect accumulation of effector leukocyte subsets like granulocytes and macrophages, local inflammation or organ injury.

Our results in the B6lpr mice with SLE and lupus nephritis do not replicate previous findings on the function of ACKR2 in other renal disease models. For example, Ackr2-deficient mice developed more severe renal injury, inflammation and fibrosis in nephrotoxic nephritis, a model of progressive immune complex-mediated glomerulonephritis which is induced by acute glomerular deposition of nephritogenic antibodies (15). Similarly, kidney injury, inflammation, and fibrotic remodeling was aggravated in Ackr2-deficient mice following severe ischemia reperfusion injury or exposure to aristolochic acid (27). In these studies disease aggravation in mice lacking Ackr2 was associated with increased renal chemokine levels and leukocyte infiltration. Thus, Ackr2-dependent chemokine clearance is apparently essential in down-regulating renal chemokine levels and inflammatory activity during the regeneration phase which follows severe but temporarily limited kidney injury. Of note, renal injury was relatively mild at week 28 in this B6lpr model of SLE, despite prominent lymphoproliferative disease which precluded an extended observational period. Thus, Ackr2-dependent chemokine scavenging activity may be sufficiently compensated by alternative mechanisms in continuous, slowly progressive injury with ongoing low-grade inflammation as it is present in lupus-like disease of B6lpr mice. Moreover, limited local inflammation may prevent sufficient intracellular mobilization of ACKR2 to the cell surface. The lack of post-transcriptional ACKR2 activation despite induced Ackr2 mRNA expression in kidney and lung of B6lpr mice may contribute to the limited functional role for ACKR2 that we observed in this model (8, 11). Therefore, Ackr2-deficiency did not result in increased inflammation and organ injury in these SLE mice. However, our data do not rule out a potential protective role of ACKR2 in more advanced lupus nephritis with more severe inflammatory kidney injury, similar to our previous findings in the nephrotoxic serum nephritis model of immune complex-mediated glomerulonephritis (15). Moreover, ACKR2`s potential to control inflammatory responses may differ depending on the underlying mechanisms of autoimmune injury in different SLE models. As such, lack of ACKR2 may result in different outcomes in other lupus prone mouse strains than B6lpr mice. In addition, this study may have been underpowered to observe minor differences in the extent of renal inflammation between WT- and Ackr2-deficient B6lpr mice, but the biologic significance of such findings might be limited.

In conclusion, this study suggests that ACKR2-mediated systemic chemokine clearance may limit formation of tertiary lymphoid tissue, but has no major role in controlling autoimmune activity or inflammatory organ injury including lupus nephritis in lupus-prone B6lpr mice. Thus, in contrast to previous work that identified a role of AKCR2 for limiting renal disease following acute episodes of injury its organ protective potential in chronic autoimmune disease may be limited.

## Data availability statement

The original contributions presented in the study are included in the article. Further inquiries can be directed to the corresponding author.

## Ethics statement

The animal study was approved by Government of Oberbayern, Germany. The study was conducted in accordance with the local legislation and institutional requirements.

## Author contributions

WX: Conceptualization, Data curation, Formal analysis, Investigation, Methodology, Validation, Writing – original draft, Writing – review & editing. NE: Data curation, Investigation, Methodology, Writing – original draft, Writing – review & editing. VV: Conceptualization, Data curation, Formal analysis, Funding acquisition, Investigation, Methodology, Project administration, Resources, Validation, Writing – original draft, Writing – review & editing.
