# Supplementary material for: The atypical chemokine receptor 2 reduces T cell expansion and tertiary lymphoid tissue but does not limit autoimmune organ injury in lupus-prone B6lpr mice
Source: Front Immunol. 2024 May 10;15:1377913. doi: 10.3389/fimmu.2024.1377913 (PMC11116673; doi:10.3389/fimmu.2024.1377913)
Supplement: Supplementary file 1 [file Image_1.pdf]

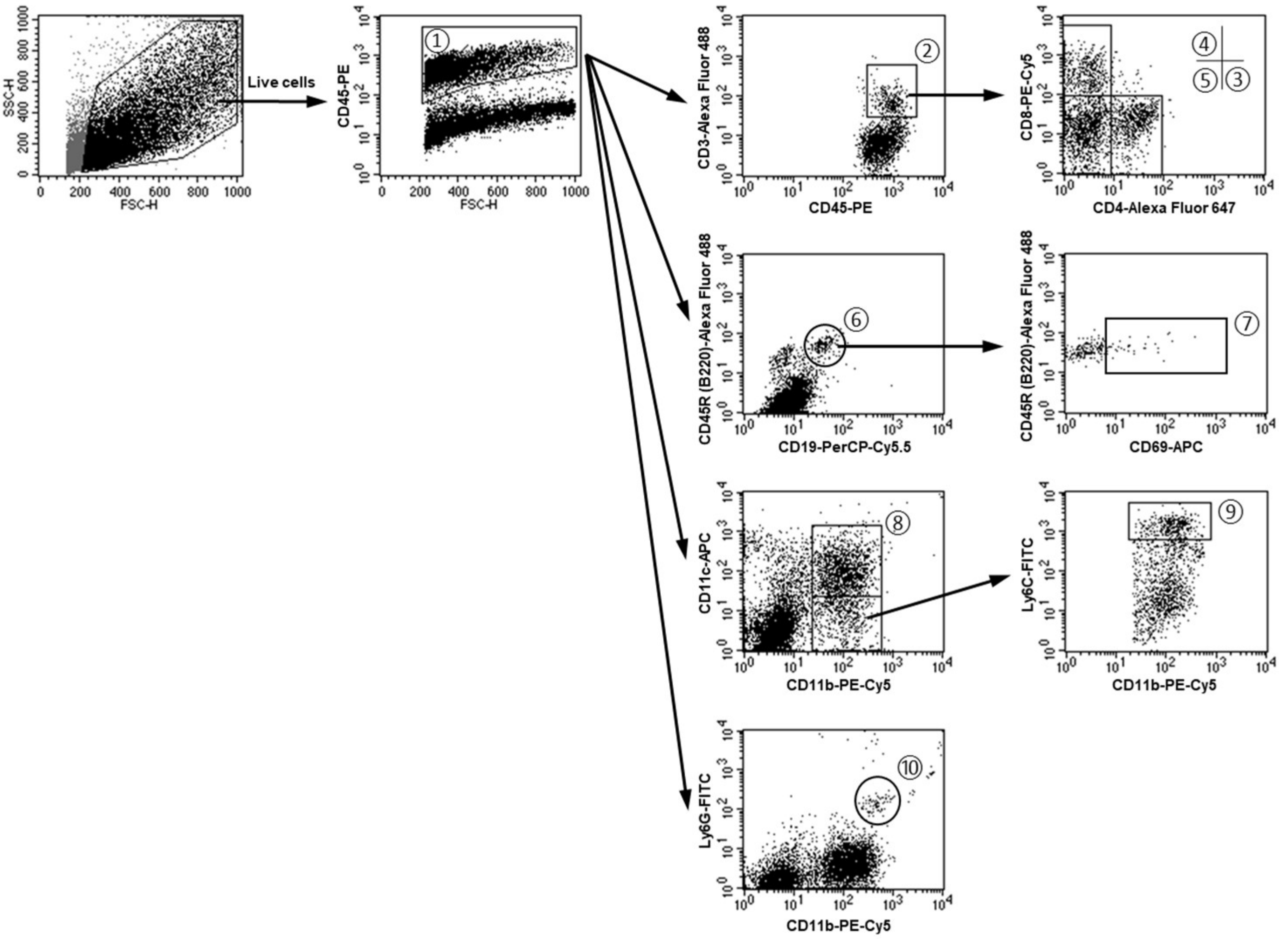

Supplementary Figure 1. Gating strategy for flow cytometry analysis of renal leukocyte populations. Representative dot blots illustrate gating of renal CD45$^+$ leukocytes (1), CD45$^+$ CD3$^+$ T lymphocytes (2), CD45$^+$ CD3$^+$ CD4$^+$ T helper cells (3), CD45$^+$ CD3$^+$ CD8$^+$ cytotoxic T cells (4), CD45$^+$ CD3$^+$ CD4$^-$ CD8$^-$ double negative T cells (5), CD45$^+$ B220$^+$ CD19$^+$ B lymphocytes (6), CD69$^+$ activated B lymphocytes (7), CD45$^+$ CD11b$^+$ CD11c$^+$ dendritic cells (8), CD45$^+$ CD11b$^+$ CD11c$^-$ Ly6C^{high} inflammatory macrophages (9), and CD45$^+$ CD11b$^+$ Ly6G$^+$ neutrophils (10).
