# Supplementary material for: The atypical chemokine receptor 2 reduces T cell expansion and tertiary lymphoid tissue but does not limit autoimmune organ injury in lupus-prone B6lpr mice
Source: Front Immunol. 2024 May 10;15:1377913. doi: 10.3389/fimmu.2024.1377913 (PMC11116673; doi:10.3389/fimmu.2024.1377913)
Supplement: Supplementary file 2 [file Image_2.pdf]

A

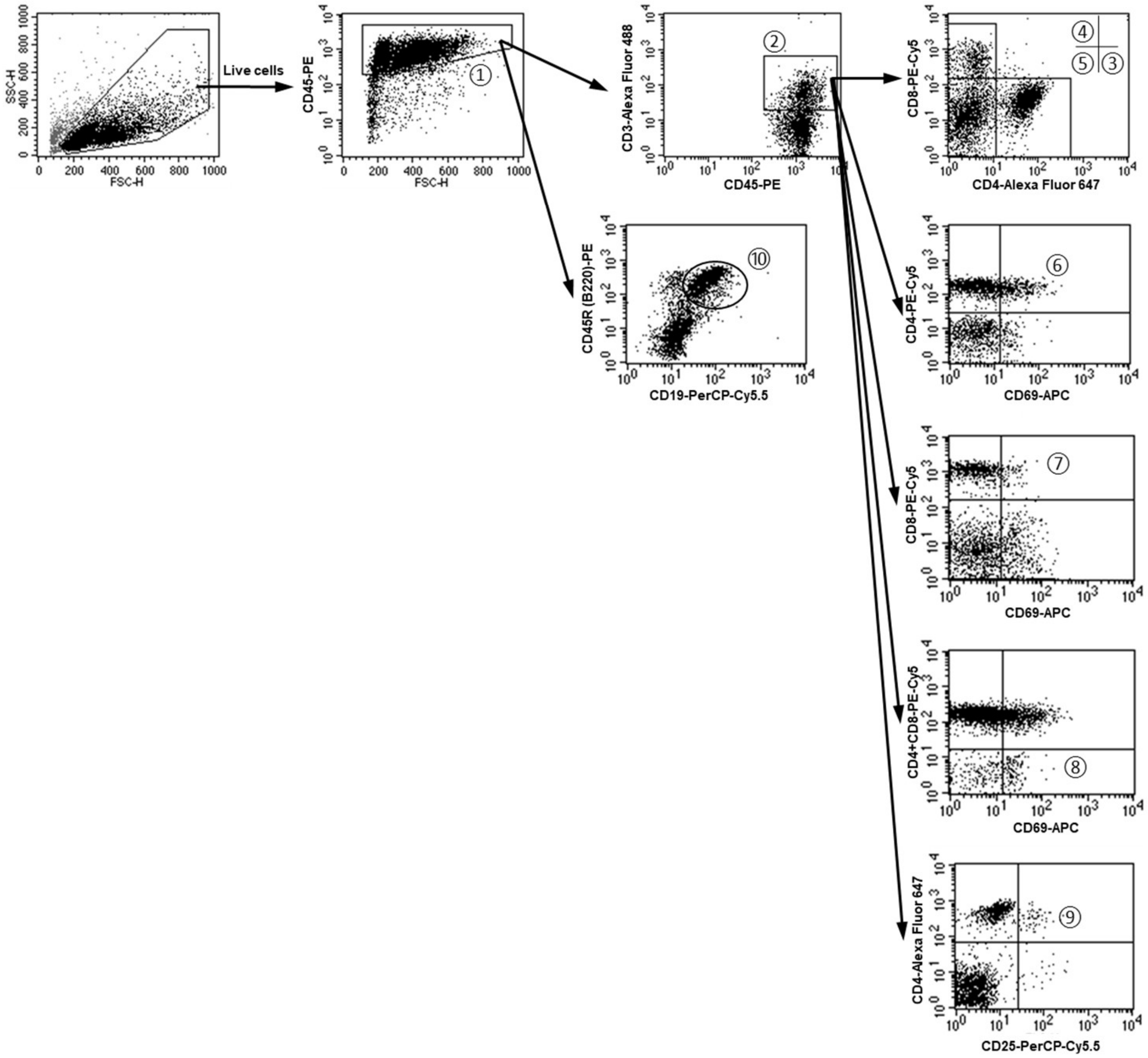

Supplementary Figure 2. Gating strategy for flow cytometry analysis of leukocyte populations in spleen and lymph nodes. **(A)** Representative dot blots illustrate gating of CD45$^+$ leukocytes (1), CD45$^+$ CD3$^+$ T lymphocytes (2), CD45$^+$ CD3$^+$ CD4$^+$ T helper cells (3), CD45$^+$ CD3$^+$ CD8$^+$ cytotoxic T cells (4), CD45$^+$ CD3$^+$ CD4$^-$ CD8$^-$ double negative T cells (5), CD 69$^+$ activated T helper cells (6), CD69$^+$ activated cytotoxic T cells (7), CD69$^+$ activated double negative T cells (8), CD45$^+$ CD3$^+$ CD4$^+$ CD24$^+$ regulatory T cells (9), and CD45$^+$ B220$^+$ CD19$^+$ B lymphocytes (10).

B

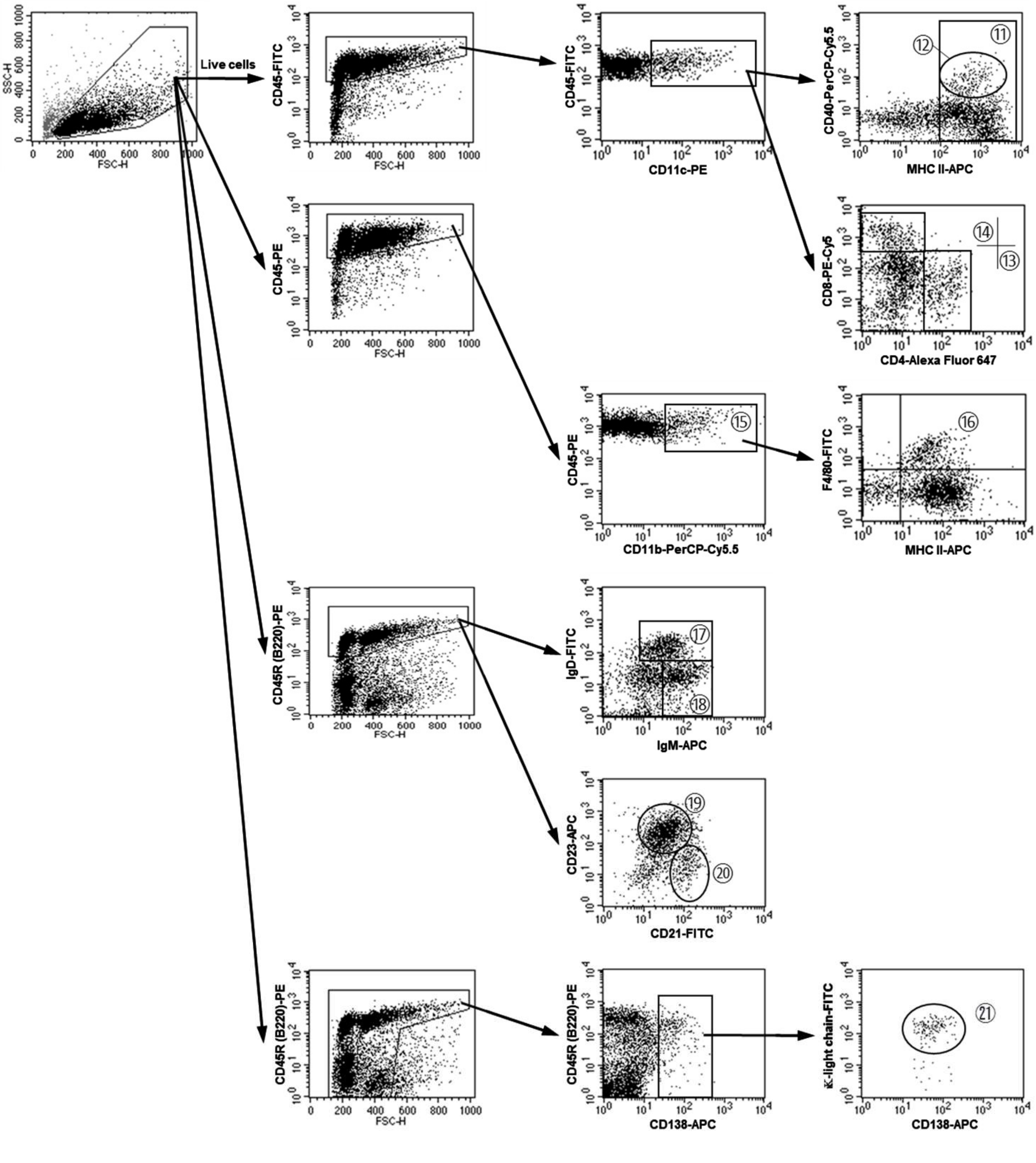

Supplementary Figure 2 (cont.). Gating strategy for flow cytometry analysis of leukocyte populations in spleen and lymph nodes. **(B)** Representative dot blots illustrate gating of CD45$^+$ CD11c$^+$ MHC II$^+$ dendritic cells (11), CD40$^+$ activated dendritic cells (12), and CD4$^+$ (13) and CD8$^+$ (14) dendritic cell subpopulations. In addition, CD45$^+$ CD11b$^+$ myeloid leukocytes (15), CD45$^+$ CD11b$^+$ MHC II$^+$ F4/80$^+$ macrophages (16), B220$^+$ IgM$^+$ IgD$^+$ mature B cells (17), B220$^+$ IgM$^+$ IgD$^-$ transitional B cells (18), B220$^+$ CD21^{low} CD23^{high} follicular B cells (19), B220$^+$ CD21^{high} CD23^{low} marginal zone B cells (20), and B220$^{+/-}$ CD138$^+$ κ-light chain$^+$ plasma cells (21) were quantitated.
