# Supplementary material for: The atypical chemokine receptor 2 reduces T cell expansion and tertiary lymphoid tissue but does not limit autoimmune organ injury in lupus-prone B6lpr mice
Source: Front Immunol. 2024 May 10;15:1377913. doi: 10.3389/fimmu.2024.1377913 (PMC11116673; doi:10.3389/fimmu.2024.1377913)
Supplement: Supplementary file 3 [file Image_3.pdf]

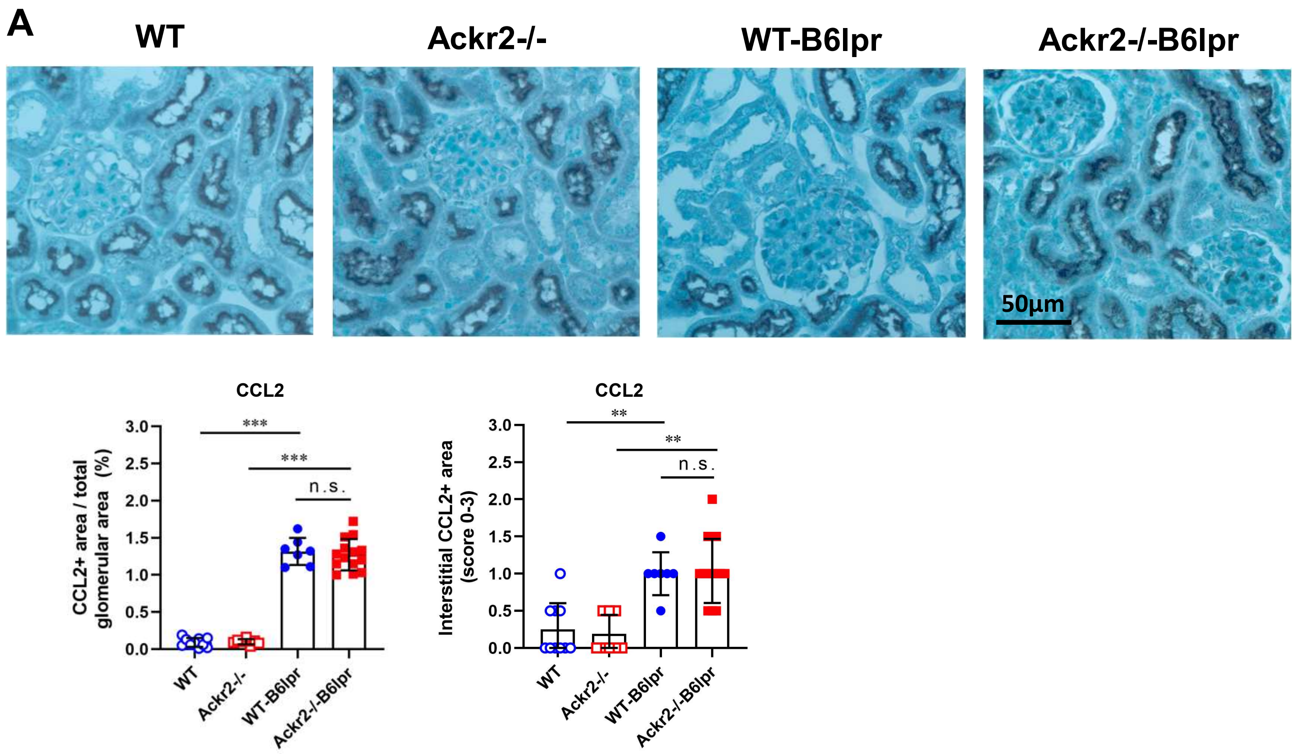

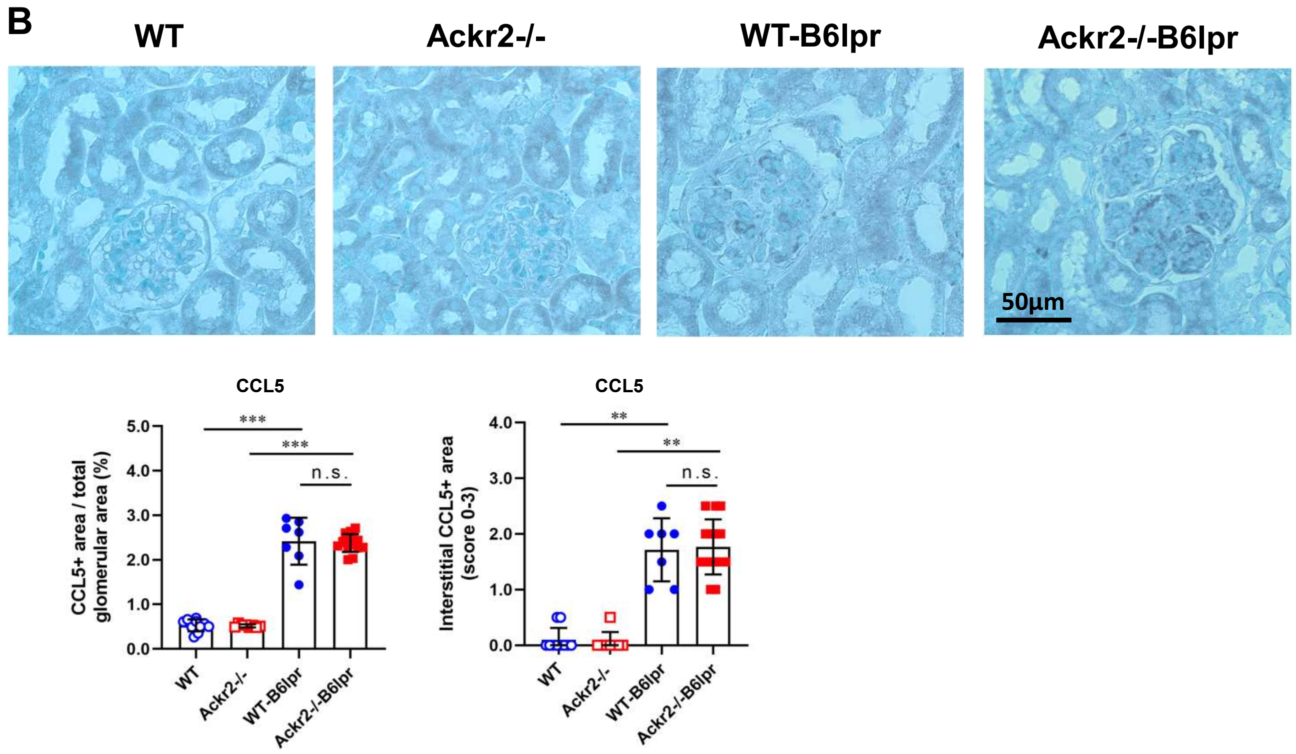

Supplementary Figure 3. Immunohistochemistry for renal **(A)** CCL2 and **(B)** CCL5. Renal sections of WT and Ackr2-/- control mice, and WT- and Ackr2-/- B6lpr mice at week 28 of lupus nephritis were stained. Representative images of glomeruli with adjacent interstitial tissue (original magnification x400) are shown. Morphometric analysis was performed as described in the Materials and methods. Data represent mean ± SD of 7 to 14 mice per group. **p<0.01; ***p<0.001; n.s., not significant.
