# Supplementary material for: The atypical chemokine receptor 2 reduces T cell expansion and tertiary lymphoid tissue but does not limit autoimmune organ injury in lupus-prone B6lpr mice
Source: Front Immunol. 2024 May 10;15:1377913. doi: 10.3389/fimmu.2024.1377913 (PMC11116673; doi:10.3389/fimmu.2024.1377913)
Supplement: Supplementary file 5 [file Image_5.pdf]

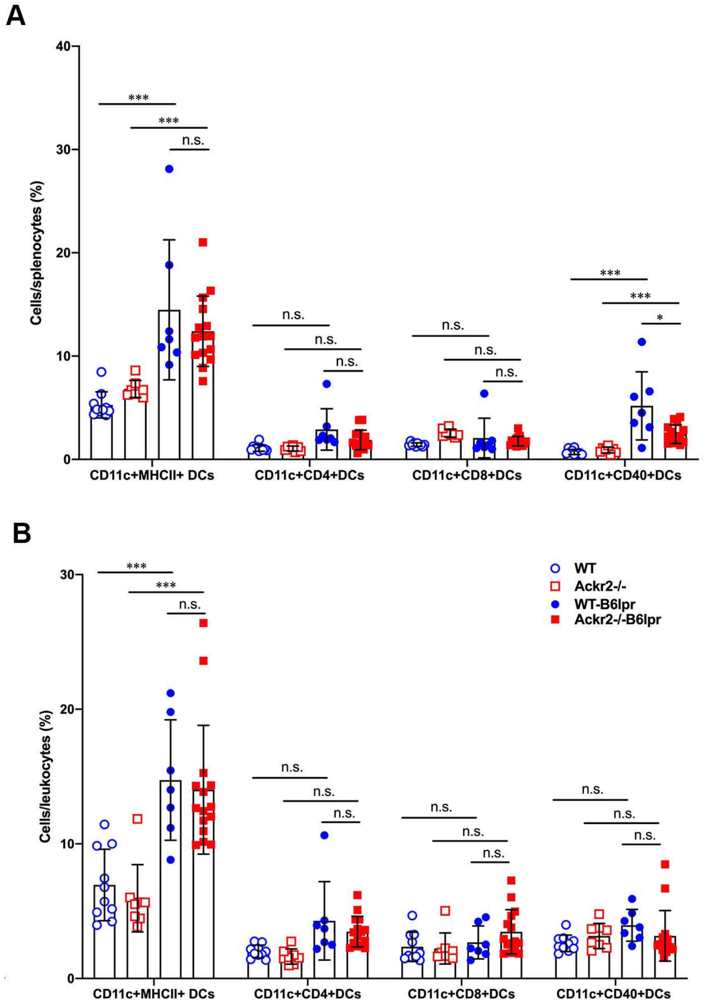

Supplementary Figure 5. Effect of Ackr2 deficiency on the relative abundance of dendritic cells (DCs) in spleens and lymph nodes of female B6lpr mice at week 28. The proportion of DC subsets in **(A)** spleen and **(B)** lymph nodes of WT and Ackr2-/- control mice, and WT- and Ackr2-/- B6lpr mice were quantified by flow cytometry demonstrating similar relative numbers in B6lpr mice of both genotypes, with the exception of a reduced relative abundance of activated CD11c^{+} CD40^{+} DCs in Ackr2-/- B6lpr spleens. Data represent mean ± SD of 7 to 15 mice per group. **p*<0.05; ****p*<0.001; n.s., not significant.
