# Supplementary material for: The atypical chemokine receptor 2 reduces T cell expansion and tertiary lymphoid tissue but does not limit autoimmune organ injury in lupus-prone B6lpr mice
Source: Front Immunol. 2024 May 10;15:1377913. doi: 10.3389/fimmu.2024.1377913 (PMC11116673; doi:10.3389/fimmu.2024.1377913)
Supplement: Supplementary file 6 [file Image_6.pdf]

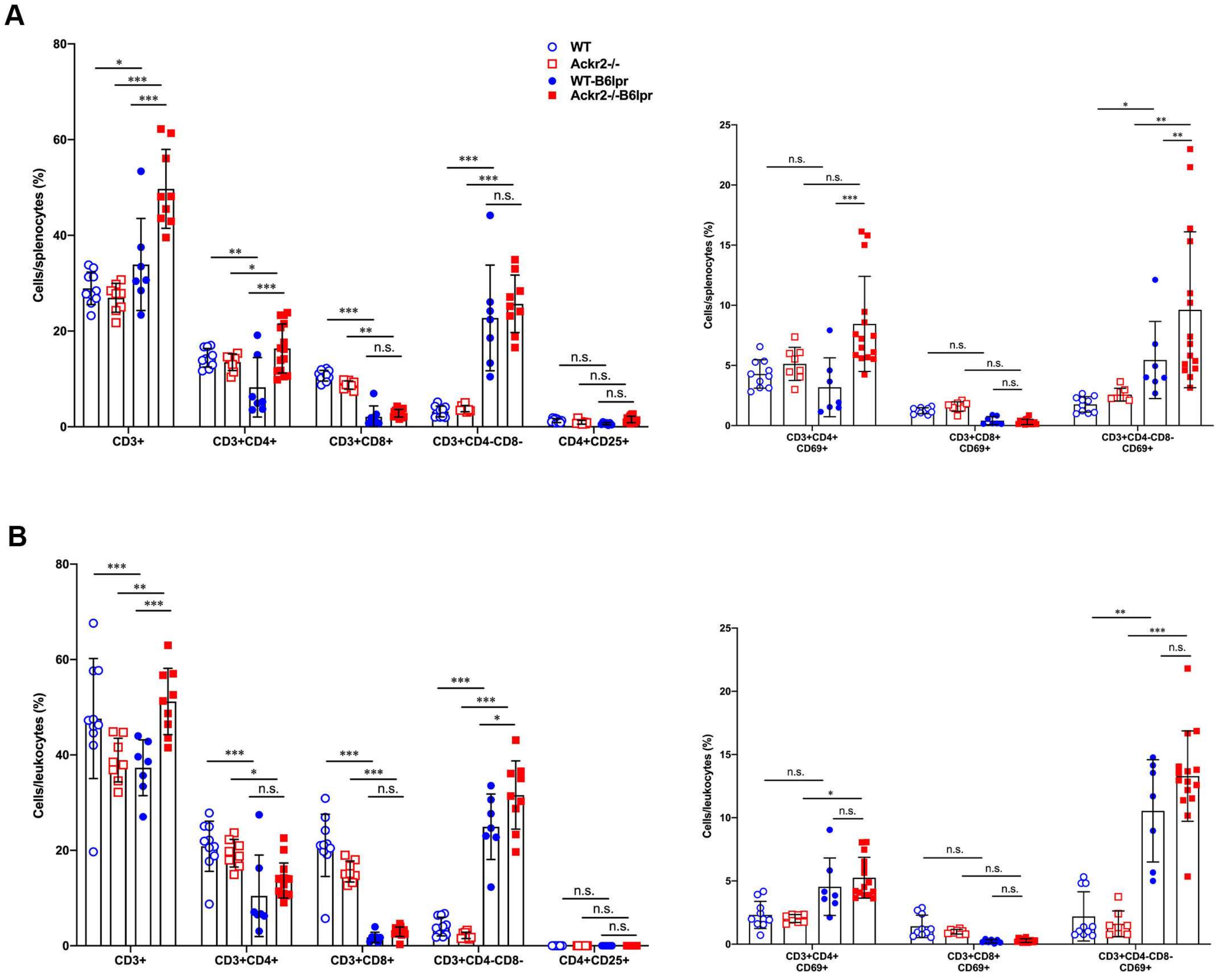

Supplementary Figure 6. Effect of Ackr2 deficiency on the relative abundance of T cells in spleen and lymph nodes in female B6lpr mice at week 28. The proportion of T cell subsets and CD69+ activated T cells were quantified by flow cytometry of **(A)** spleens and **(B)** lymph nodes isolated from WT and Ackr2-/- control mice, and WT- and Ackr2-/- B6lpr mice. Compared to WT-B6lpr mice Ackr2-/- B6lpr mice showed significantly increased relative numbers of CD3^{+} CD4^{+} T cells and activated CD69^{+} CD3^{+} CD4^{+} T cells in spleens, and increased relative numbers of CD3^{+} T cells and CD3^{+} CD4^{-} CD8^{-} double negative T cells in lymph nodes. Data represent mean ± SD of 7 to 15 mice per group. *$p<0.05$; **$p<0.01$; ***$p<0.001$; n.s., not significant.
