# Supplementary material for: The atypical chemokine receptor 2 reduces T cell expansion and tertiary lymphoid tissue but does not limit autoimmune organ injury in lupus-prone B6lpr mice
Source: Front Immunol. 2024 May 10;15:1377913. doi: 10.3389/fimmu.2024.1377913 (PMC11116673; doi:10.3389/fimmu.2024.1377913)
Supplement: Supplementary file 4 [file Image_4.pdf]

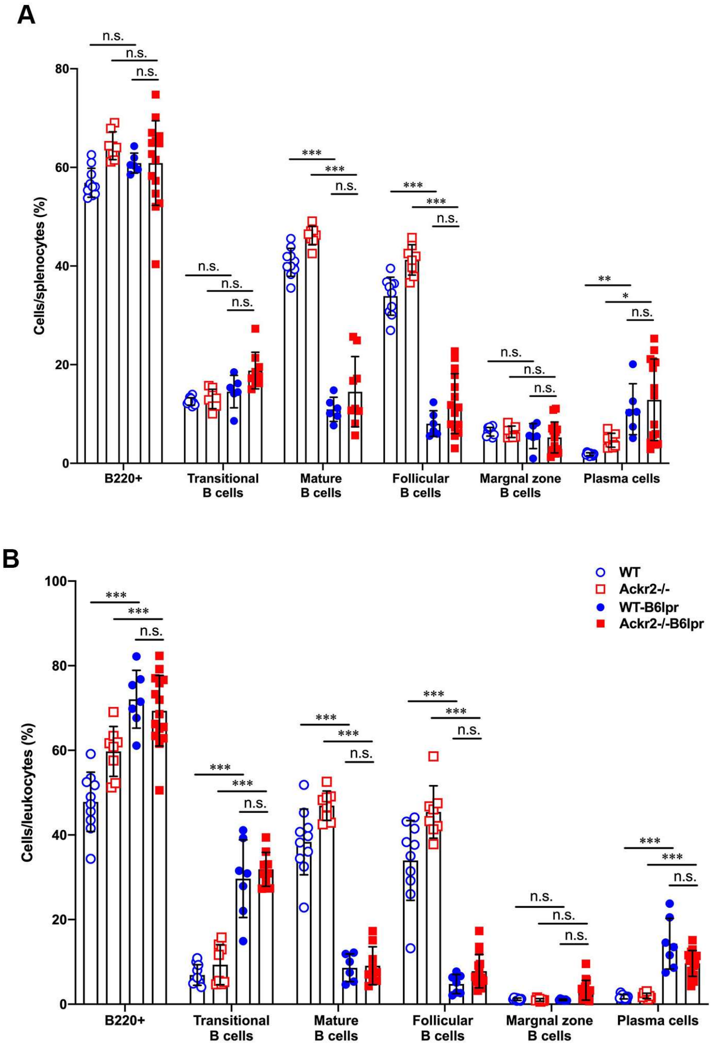

Supplementary Figure 4. Effect of Ackr2 deficiency on the relative abundance of B cell populations in female B6lpr mice at week 28. The proportion of **(A)** spleen and **(B)** lymph node B cell subsets were quantitated by flow cytometry identifying B220^{+} IgM^{+} transitional B cells, B220^{+} IgM^{+} IgD^{+} mature B cells, B220^{+} CD21^{high} CD23^{low} marginal zone B cells, B220^{+} CD21^{low} CD23^{high} follicular B cells, and B220$^{+/-}$ CD138^{+} k-light chain^{+} plasma cells and revealed similar relative numbers in WT- and Ackr2-/- B6lpr mice. Data represent mean ± SD of 7 to 15 mice per group. *p<0.05; **p<0.01; ***p<0.001; n.s., not significant.
